# Inhibition of Rho Kinase Induces Antioxidative Molecules and Suppresses Reactive Oxidative Species in Trabecular Meshwork Cells

**DOI:** 10.1155/2017/7598140

**Published:** 2017-07-19

**Authors:** Tomokazu Fujimoto, Toshihiro Inoue, Saori Ohira, Nanako Awai-Kasaoka, Takanori Kameda, Miyuki Inoue-Mochita, Hidenobu Tanihara

**Affiliations:** ^1^Department of Ophthalmology, Faculty of Life Sciences, Kumamoto University, 1-1-1 Honjo, Kumamoto 860-8556, Japan; ^2^Department of Ophthalmology and Visual Sciences, Kyoto University Graduate School of Medicine, Yoshida-Konoe-cho, Sakyo-ku, Kyoto 606-8501, Japan

## Abstract

**Purpose:**

To investigate the effect of rho kinase inhibitors on oxidative stress in trabecular meshwork (TM) cells.

**Methods:**

TM cells were isolated from the eyes of cynomolgus monkeys. Y-27632 and menadione were used to inhibit rho kinase and induce production of reactive oxygen species (ROS), respectively. The cynomolgus monkey array and 12,613 probes were used in DNA microarray analysis, and the affected genes were categorized using gene ontology analysis. The mRNA levels of the target genes were confirmed by real-time RT-PCR. Intracellular oxidative stress was detected using a fluorescent reagent sensitive to ROS. Cell viability was assessed by the WST-8 assay.

**Results:**

Gene ontology analysis revealed upregulation of genes involved in antioxidant activity, and upregulation of catalase was confirmed by real-time RT-PCR after 30 min treatment with Y-27632. Production of ROS was increased by menadione, and the effect was partly suppressed by pretreatment with Y-27632. At a lower dose of menadione, Y-27632 stimulated TM cells and significantly increased their viability following menadione treatment compared to control cells.

**Conclusion:**

Using microarray analysis, Y-27632 was shown to upregulate antioxidative genes including catalase and partially reduce ROS production and cell death by oxidative stress caused by menadione.

## 1. Introduction

Oxidative stress is a major physiological phenomenon, mediated through the production of reactive oxygen species (ROS), such as peroxides, superoxide, hydroxyl radical, and singlet oxygen. ROS play an important role in cell homeostasis and pathogen response and are therefore essential in biological processes. In contrast, increases in ROS are seen in various age-related diseases including glaucoma [1]. For instance, in the aqueous humor of glaucoma patients, the levels of oxidative stress markers are significantly increased [2–5]. Additionally, oxidative DNA damage is reportedly increased in the trabecular meshwork (TM) of glaucoma patients [6, 7]. These findings indicate that the TM of glaucomatous eyes is continuously exposed to oxidative stress, and therefore, damage to TM may increase outflow resistance and the risk of glaucoma progression. In line with this, lower systemic antioxidant capacity is related to higher intraocular pressure (IOP) levels in open-angle glaucoma patients [8]. Moreover, glaucoma-related genes, such as *CYP1B1* and *FOXC1*, are reportedly linked to oxidative stress in the eyes [9–12]. Taken together, control of oxidative stress in the eye may be a therapeutic target to slow glaucoma progression.

Rho-rho kinase (ROCK) signaling controls polymerization of actin and thereby mediates various cell functions, such as contraction, migration, phagocytosis, and mitosis. Inhibition of ROCK increases aqueous outflow by depolymerizing F-actin in TM cells and Schlemm's canal endothelial cells [13, 14]. A ROCK inhibitor, ripasudil, has been approved as an IOP-lowering drug in Japan [15]. Ripasudil significantly reduces the IOP of glaucoma patients upon either single or multiple administration [16, 17]. However, ROCK inhibitors have drawn attention as antioxidative drugs against cardiovascular diseases and chronic renal injury [18, 19]. Indeed, ripasudil (also known as K-115) has been reported to have a neuroprotective effect on the optic nerve by suppressing oxidative stress in an animal model [20]. Thus, the effect of ROCK inhibitors on oxidative stress in TM cells is of interest from a therapeutic point of view against glaucoma.

Here, we show the results of an exhaustive investigation using a microarray, revealing that treatment with Y-27632, a well-known ROCK inhibitor, upregulates antioxidative molecules in TM cells, inhibits ROS production, and promotes cell survival.

## 2. Materials and Methods

### 2.1. Cell Culture

Trabecular meshwork (TM) cells were isolated from the eyes of cynomolgus monkeys (Shin Nippon Biomedical Laboratories, Kagoshima, Japan) according to the method described previously [21]. Primary TM cells were cultured in Dulbecco's modified Eagle's medium (DMEM; Wako, Osaka, Japan) supplemented with 10% FBS, 2 mM glutamine, 100 U/mL penicillin, 100 *μ*g/mL streptomycin, and 0.5 *μ*g/mL amphotericin B at 37°C in 5% CO_2_. These cells were used after 2–5 passages. The character of the isolated cells in the present study was confirmed by expression of specific TM markers (caveolin 1, collagen 4*α*5, matrix gla protein, tissue inhibitor of metalloproteinase 3, and vascular cell adhesion protein 1), phagocytosis function, and myocilin induction by dexamethasone as described previously [22].

### 2.2. DNA Microarray Analysis

Custom cDNA microarray analysis was performed using a CombiMatrix microarray (CombiMatrix, Mukilteo, WA) as described previously [23]. Briefly, the cynomolgus monkey array was designed to detect directly labeled mRNA from 12,613 probes. Confluent TM cells in 100 mm dishes were treated with 25 *μ*M Y-27632 (Merck Millipore, Darmstadt, Germany) or vehicle (deionized water) for 30 min. Total RNA was extracted from the cells, and the integrity and concentration of total RNA was measured using an Agilent 2100 Bioanalyzer (Agilent Technologies, Santa Clara, CA). Fluorescence-labeled antisense RNA was synthesized by direct incorporation of Cy5-UTP or Cy3-UTP, using each RNA sample and an RNA Transcript SureLABEL Core kit (Takara Bio, Shiga, Japan). Labeled antisense RNAs were hybridized simultaneously with the microarray chips. DNA microarray preparation, hybridization, processing, scanning, and analyses were performed according to the manufacturer's instructions (Filgen, Nagoya, Japan). Fluorescent images of hybridized microarrays were obtained with a GenePix 4000B Scanner (Molecular Devices, Sunnyvale, CA). Array-Pro Analyzer Ver4.5 (Media Cybernetics, Silver Spring, MD) was used to determine the signal intensity of each spot and its local background. Scanned images were analyzed using Microarray Data Analysis Tool Ver3.2 software (Filgen). Signals from Y-27632 treated cells were compared with those from vehicle-treated cells, and genes that showed greater than 3/2-fold change in expression in at least one of the pairwise probe comparisons were considered upregulated, whereas those that showed a change of expression smaller than 2/3-fold were considered downregulated. These analyses were performed three times using TM cells from three different monkeys independently, and genes with common differences in expression among the three experiments were identified as affected genes. The affected genes were further analyzed by gene ontology, in which putative functions of gene products were categorized as “biological process,” “cellular component,” or “molecular function” by a BLAST homology search of EST sequences available from the National Center for Biotechnology Information.

### 2.3. Real-Time RT-PCR

Total RNA was isolated from cultured TM cells treated with Y-27632 for 30 min using NucleoSpin RNA (Macherey-Nagel, Düren, Germany). Total RNA was reverse transcribed (PrimeScript RT Master Mix; Takara Bio Inc., Shiga, Japan) according to the manufacturer's protocol. Quantitative real-time RT-PCR was performed using an ABI Prism 7000 (Life Technologies). Reactions were performed in 20 *μ*L of reaction mixture containing 10 *μ*L PCR master mix (SYBR Premix Ex Taq II; Takara Bio Inc.), 0.4 *μ*M primer pairs, and 2 *μ*L cDNA samples. The gene-specific primer pairs were as follows: monkey catalase, forward (F) 5′-GCA AAT CTG TGA GGC CGG GG-3′; reverse (R) 5′-GCG CAT CTA GCA CCG GAG AA-3′ and 18S ribosomal RNA, (F) 5′-GCC CGA AGC GTT TAC TTT GA-3′; (R) 5′-CCG CGG TCC TAT TCC ATT ATT-3′. The thermal cycling conditions were 95°C for 30 s and 40 cycles of 95°C for 5 s and 60°C for 31 s. All PCR reactions were performed in duplicate.

Relative expression of catalase in the Y-27632-treated samples was compared to that in control samples using the comparative C_T_ method (ΔΔC_T_ method); 18S ribosomal RNA was used as an endogenous control. The threshold cycle, C_T_, was determined after setting the threshold in the linear amplification phase of the PCR reaction and ΔC_T_ was defined as ΔC_T_ = C_T_ (target gene) − C_T_ (18S rRNA). Relative expression of the target gene was calculated as: 2^−ΔΔCT^, ΔΔC_T_ = ΔC_T_ (treated sample) − ΔC_T_ (control).

### 2.4. Intracellular Oxidative Stress Detection

The effects of Y-27632 on the production of ROS were evaluated using CellROX® green reagent (Life Technologies) in the TM cells. These cells were cultured on 6 cm dishes in DMEM containing 10% FBS and antibiotics at 37°C in 5% CO_2_. After cells had grown to confluence, they were pretreated with Y-27632 for 30 min and then stimulated with 100 *μ*M menadione (Sigma, St. Louis, MO) for 1 h. CellROX reagent was then added to each dish to give a final concentration of 5 *μ*M and incubated for 30 min at 37°C. After incubation, TM cells were washed in PBS and detached by trypsin/EDTA solution and centrifuged at 1200 rpm for 3 min. The supernatant was removed, and cells were fixed in 4% paraformaldehyde in PBS for 15 min and then centrifuged twice at 1200 rpm for 3 min, resuspending in PBS after each spin. FITC fluorescence of TM cells was analyzed using a Cell Sorter SH800 (Sony Biotechnology, Tokyo, Japan).

### 2.5. Cell Viability Assay

The effects of Y-27632 on TM cell viability were evaluated using the WST-8 assay (Cell Counting Kit-8, Dojindo Laboratories, Kumamoto, Japan). Cells were seeded on 96-well plates (1 × 10^4^ cells/well) and incubated at 37°C under 5% CO_2_ overnight. After pretreatment with Y-27632 for 30 min, cells were stimulated with H_2_O_2_ or menadione for 24 h. CCK-8 reagents were added into each well and incubated for 2 h at 37°C. Absorbance at 450 nm was determined using a microplate reader (Multiskan FC, Thermo Fisher Scientific). Cell viability was expressed as a percentage of control (vehicle-treated) cells.

### 2.6. Direct Antioxidant Activity of Y-27632

Direct antioxidant activity was assessed by 2-methyl-6-p-methoxyphenylethynylimidazopyrazinone (AB-2950 MPEC; ATTO, Tokyo, Japan), a superoxide-sensitive luminescent reagent, and reagents for xanthine-oxidase-induced superoxide production (AB-2970 CLETA-S, ATTO) following the manufacturer's protocol. Briefly, 10 *μ*L of 300 *μ*M MPEC/ethanol and 80 *μ*L of 1.25 unit/mL xanthine oxidase/HEPES were mixed. Then, 10 *μ*L of 25 *μ*M Y-27632 or 20 mM n-acetyl cysteine (positive control) was added into each well of a 96-well plate. Subsequently, 90 *μ*L of the mixture of MPEC and xanthine oxidase and 200 *μ*L of xanthine were added to each well. The luminescent signal was measured for 10 s by a luminometer (AB-2270 Octa; ATTO).

### 2.7. Statistical Analysis

Data are presented as means ± standard error. Statistical comparisons of multiple groups were performed using the Tukey-Kramer HSD test and Dunnett's test, and those of two groups were performed using Wilcoxon rank sum test and Wilcoxon signed rank test. Differences were considered statistically significant at *P* < 0.05.

## 3. Results

### 3.1. Microarray Expression Profile in Y-27632-Treated TM Cells

**Figure 1:**
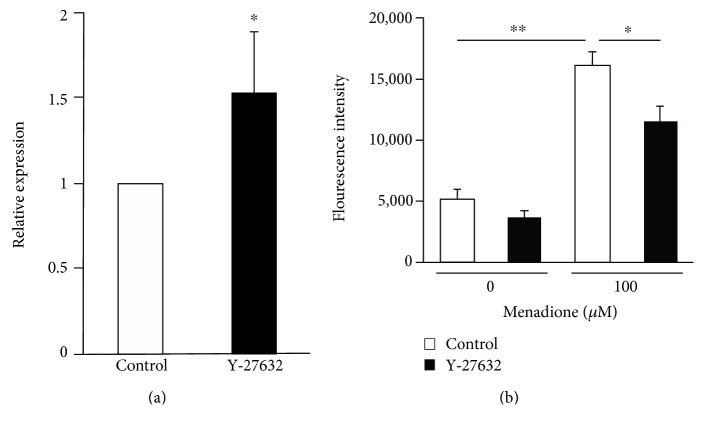
(a) Quantitative PCR analysis of catalase mRNA. The TM cells were treated with 25 *μ*M Y-27632 for 30 min. The relative expression level of *catalase* of samples treated with Y-27632 was compared to that of the control sample using the comparative C_T_ method (ΔΔC_T_ method). The 18S ribosomal RNA was used as an endogenous control. Data are shown as mean ± SE from six independent experiments. ^∗^*P* < 0.05 compared with control by Wilcoxon rank sum test. (b) The effects of Y-27632 on the intracellular production of reactive oxygen species (ROS). The TM cells were treated with or without 25 *μ*M Y-27632 for 30 min, followed by 100 *μ*M menadione stimulated for 1 h. ROS were detected by CellROX reagent, and the fluorescence of the TM cells were measured by cell sorter SH800. Data are shown as mean ± SE from five independent experiments. ^∗∗^*P* < 0.01 and ^∗^*P* < 0.05 compared with control by the Wilcoxon rank sum test (a) and Tukey Kramer HSD test (b).

Among the 12,613 genes analyzed by microarray, the affected genes are listed in Tables 1 and 2; 444 genes were upregulated, and 56 were downregulated. Significantly upregulated and downregulated gene categories based on gene ontology analysis in Y-27632 treated TM cells are listed in Tables 3 and 4. Gene ontology analysis revealed that the upregulated genes were related to various cellular functions including antioxidant activity (*P* = 0.014), and downregulated genes were related to integrin complexes (*P* = 0.039), and calcium ion transport into the cytosol (*P* = 0.008). In the category of antioxidant activity, upregulated genes were homologous to human gene coding catalase (*P* = 0.046), thioredoxin domain-containing 2 (also known as spermatozoa; *P* = 0.032), nucleoredoxin (*P* = 0.017), albumin (probe 1, *P* = 0.002; probe 2, *P* = 0.021), and glutathione transferase zeta 1 (*P* = 0.004). Upregulation of the mRNA of catalase, an extensively investigated antioxidant, was confirmed by real-time RT-PCR and found to be 1.5 times higher in TM cells treated with Y-27632 compared to the control TM cells (*P* = 0.032; Figure 1(a)). In contrast, four other genes involved in antioxidant activity were not significantly affected after treatment with Y-27632 (data not shown).

### 3.2. Effects of Y-27632 on the Production of Reactive Oxygen Species in TM Cells

To assess the effects of Y-27632 on the production of ROS in TM cells, we utilized a fluorogenic probe that exhibits bright fluorescence upon oxidation by ROS. In the absence of an oxidative reagent, the fluorescence intensity was not significantly different in TM cells treated with Y-27632 compared to control (3673.2 ± 452.3 versus 5104.5 ± 735.0; Figure 1(b)). In the presence of 100 *μ*M menadione, the fluorescence intensity was significantly elevated (16097.7 ± 1133.0; *P* < 0.0001); this elevation was partly suppressed by treatment with Y-27632 (11443.6 ± 1332.2; *P* = 0.0182), suggesting that Y-27632 reduces ROS production in TM cells under oxidative stress.

### 3.3. Effects of Y-27632 on the Viability of TM Cells under Oxidative Stress

**Figure 2:**
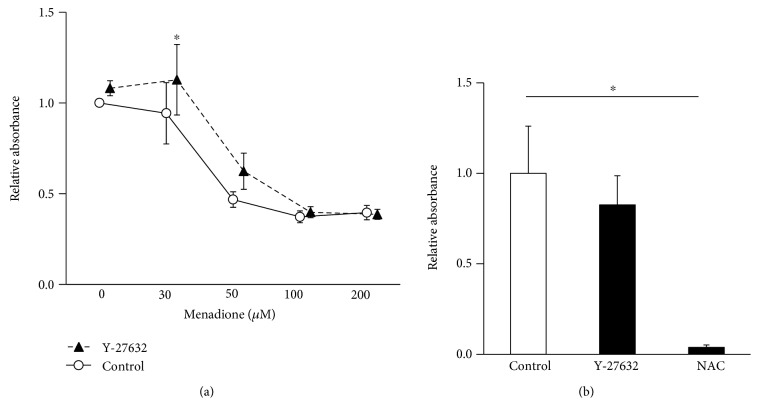
(a) The effect of Y-27632 on oxidative stress-induced cell death. The TM cells were treated with or without 25 *μ*M Y-27632 for 30 min, followed by menadione stimulation of the cells for 24 h. Cell viabilities were shown as relative value compared with the control. Data are shown as the mean ± SE from six independent experiments. ^∗^*P* < 0.05 compared with control by Wilcoxon rank sum test. (b) The effect of Y-27632 on extracellular antioxidative activity. The xanthine-oxidase-induced superoxide production was assessed using a superoxide-sensitive luminescent reagent. Data are shown as the mean ± SE from six independent experiments. ^∗^*P* < 0.05 compared with the control by Dunnett's test. Nac: n-acetyl cysteine.

Finally, we investigated the effects of Y-27632 on the viability of TM cells under oxidative stress. As shown in Figure 2(a), menadione reduced TM cell viability in a dose-dependent manner. At a lower dose of menadione, Y-27632-stimulated TM cells regained significant viability against menadione treatment compared to control cells (*P* = 0.0238). In contrast, the effects of Y-27632 on cell viability were not significant at a higher dose of menadione.

### 3.4. Direct Antioxidant Activity of Y-27632

To confirm the extracellular antioxidant activity of Y-27632, we assessed xanthine oxidase-induced superoxide production using a luminescent reagent. As shown in Figure 2(b), there was no significant difference in ROS production between the control and Y-27632 treatment. Thus, Y-27632 does not appear to affect extracellular oxidants.

## 4. Discussion

In the present study, we have identified the antioxidative effect of Y-27632 in TM cells by microarray analysis, an exhaustive investigation of gene expression, and shown that Y-27632 partially suppresses ROS production and cell death induced by menadione. To the best of our knowledge, this is the first report to show the antioxidant effect of ROCK inhibitor on TM cells. Previously, we presented depolymerization of F-actin before morphometric recovery from oxidative stress in TM cells [24], suggesting a correlation between oxidative stress and regulation of the actin cytoskeleton in TM cells. In other tissues, rho-kinase was identified as a mediator of various diseases associated with inflammation and oxidative stress, and inhibition of rho-kinase has been drawing attention as a promising therapeutic strategy. For instance, activation of the rho/rho-kinase pathway is related to the pathophysiology of chronic renal injury, and long-term fasudil treatment has renoprotective effects in this malignant hypertension model. The mechanism of the renoprotective effect of fasudil, a nonspecific ROCK inhibitor, was suggested to involve a combination of factors, including inhibition of the TGF-*β*-collagen cascade, control of inflammation, reduction of oxidative stress, and upregulation of eNOS [18]. Clinical studies with fasudil have suggested that it may be useful for the treatment of a wide range of cardiovascular diseases [19]. Importantly, rho-kinase inhibitors block ROS production by suppressing CyPA secretion from vascular smooth muscle cells [25], suggesting the beneficial effect of rho-kinase inhibitors against cardiovascular diseases.

Recently, Yamamoto and colleagues demonstrated the neuroprotective effect of the ROCK inhibitor K-115, a novel IOP-lowering drug, using the mouse optic crush model [20]. They showed the effect was at least partially dependent on suppression of ROS production via inhibition of *Nox1* expression in retinal ganglion cells. We also showed that ROCK inhibitors' antioxidant effects are indirect using monkey TM cells. However, in the present study using microarray analysis, *Nox* family genes were not identified as affected, but catalase was upregulated after treatment with Y-27632. This disagreement might be caused by differences in species and/or tissues. Thus, the precise molecular mechanisms of the antioxidative effect of ROCK inhibitors have not been clarified completely. On the other hand, a recent study reported that Y-27632 induced p-53-mediated apoptosis in hemangioma [26]. In the present study, we indicated that ROCK inhibitor effected cell survival in TM cells. This is interesting point since ROCK inhibitor-induced effects such as cell death or cell protection were changed by differences of cell types.

TM has a critical role in the maintenance of aqueous outflow resistance through the regulation of extracellular matrix metabolism, phagocytosis of debris, and empty space associated with tissue contraction [27, 28]. Indeed, the number of TM cells is decreased in glaucomatous eyes [29], suggesting that functional TM cells are essential in controlling IOP. In this context, oxidative stress is a potential cause of cellular dysregulation in TM, both functionally and numerally, because it has been suggested that the TM of glaucomatous eyes is continuously exposed to oxidative stress [2–7]. Thus, an antioxidant drug might reduce oxidative stress in TM cells, slowing progression of glaucomatous damage in outflow tissues. Though it remains unknown whether clinically used eye-drops containing ripasudil have significant antioxidative effects on TM cells in vivo, the present study's findings may be clinically relevant.

The effect of Y-27632 on cell survival under oxidative stress was significant, but limited. Since glaucoma progresses chronically in the majority of the patients, the acute oxidative damage in the present study may not reflect pathological conditions in glaucomatous TM cells, which is one of the limitations of the present study. Another limitation is that the antioxidative effects of ROCK inhibition were not corroborated in vivo. Further studies are required to acquire more clinically relevant evidence of the effects of ROCK inhibitor on oxidative stress in TM.

## 5. Conclusion

Microarray analysis reveals that Y-27632 upregulates antioxidative genes including catalase and partially reduces the ROS production and cell death by oxidative stress induced by menadione.

## Figures and Tables

**Table 1:** Genes that are upregulated in TM cells.

Accession number	Human RefSeq description	Fold change
DW528016	gi|75750485|ref|NM_004773.2 *Homo sapiens* zinc finger, HIT type 3 (ZNHIT3), mRNA	6.79928
CJ434702	gi|20986504|ref|NM_002753.2 *Homo sapiens* mitogen-activated protein kinase 10 (MAPK10), transcript variant 1, mRNA	5.85538
AB168851	gi|224586874|ref|NM_033124.4 *Homo sapiens* coiled-coil domain-containing 65 (CCDC65), mRNA	5.77453
AB169150	gi|223555972|ref|NR_026827.1 *Homo sapiens* hypothetical LOC84856 (LOC84856), noncoding RNA	5.01086
DW523643	gi|225903398|ref|NM_001146152.1 *Homo sapiens* cytochrome P450, family 51, subfamily A, polypeptide 1 (CYP51A1), transcript variant 2, mRNA	4.6977
AK240630	gi|4503754|ref|NM_002021.1 *Homo sapiens* flavin-containing monooxygenase 1 (FMO1), mRNA	4.64946
BB894083	gi|154689768|ref|NM_020840.1 *Homo sapiens* folliculin-interacting protein 2 (FNIP2), mRNA	4.52052
AB168218	gi|85060516|ref|NM_199321.2 *Homo sapiens* zona pellucida-binding protein 2 (ZPBP2), transcript variant 2, mRNA	4.20286
AB168199	gi|156523965|ref|NM_001102470.1 *Homo sapiens* alcohol dehydrogenase 6 (class V) (ADH6), transcript variant 1, mRNA	3.89514
AB172502	gi|50897849|ref|NM_001001936.1 *Homo sapiens* actin filament-associated protein 1-like 2 (AFAP1L2), transcript variant 1, mRNA	3.84241
CJ448047	gi|46909588|ref|NM_002731.2 *Homo sapiens* protein kinase, cAMP-dependent, catalytic, beta (PRKACB), transcript variant 2, mRNA	3.75324
DC857227	gi|239752603|ref|XM_002348257.1 PREDICTED: *Homo sapiens* similar to immunoglobulin lambda-like polypeptide 1 (LOC100294459), mRNA	3.66898
CJ449582	gi|9506614|ref|NM_019023.1 *Homo sapiens* protein arginine methyltransferase 7 (PRMT7), mRNA	3.64058
EF208813	gi|24797075|ref|NM_002121.4 *Homo sapiens* major histocompatibility complex, class II, DP beta 1 (HLA-DPB1), mRNA	3.57386
DQ417745	gi|194,248,050|ref|NM_000839.3 *Homo sapiens* glutamate receptor, metabotropic 2 (GRM2), transcript variant 1, mRNA	3.5509
AB049894	gi|66571326|ref|NM_020914.3 *Homo sapiens* ring finger protein 213 (RNF213), mRNA	3.51068
DC850932	gi|117676364|ref|NM_014350.2 *Homo sapiens* tumor necrosis factor, alpha-induced protein 8 (TNFAIP8), transcript variant 1, mRNA	3.47535
BB877436	gi|142360165|ref|NM_005123.2 *Homo sapiens* nuclear receptor subfamily 1, group H, member 4 (NR1H4), mRNA	3.46906
AB174726	gi|22208962|ref|NM_016150.3 *Homo sapiens* ankyrin repeat and SOCS box-containing 2 (ASB2), mRNA	3.30479
AB174122	gi|209862773|ref|NM_002483.4 *Homo sapiens* carcinoembryonic antigen-related cell adhesion molecule 6 (nonspecific cross-reacting antigen) (CEACAM6), mRNA	3.27904
AB173773	gi|38569483|ref|NM_017641.2 *Homo sapiens* kinesin family member 21A (KIF21A), mRNA	3.26679
DK578446	gi|15718677|ref|NM_033257.2 *Homo sapiens* DiGeorge syndrome critical region gene 6 like (DGCR6L), mRNA	3.26114
AB168950	gi|38261961|ref|NM_018179.3 *Homo sapiens* activating transcription factor 7-interacting protein (ATF7IP), mRNA	3.25607
CJ488707	gi|45333915|ref|NM_178456.2 *Homo sapiens* chromosome 20 open reading frame 85 (C20orf85), mRNA	3.24156
DC639327	gi|154800442|ref|NM_005074.3 *Homo sapiens* solute carrier family 17 (sodium phosphate), member 1 (SLC17A1), mRNA	3.23693
BB895966	gi|32483409|ref|NM_000583.2 *Homo sapiens* group-specific component (vitamin D-binding protein) (GC), mRNA	3.22662
DQ417744	gi|194248050|ref|NM_000839.3 *Homo sapiens* glutamate receptor, metabotropic 2 (GRM2), transcript variant 1, mRNA	3.1862
AB168486	gi|195972893|ref|NM_152764.2 *Homo sapiens* chromosome 16 open reading frame 73 (C16orf73), mRNA	3.17552
AB047624	gi|45446748|ref|NM_004984.2 *Homo sapiens* kinesin family member 5A (KIF5A), mRNA	3.1749
CJ446015	gi|187761371|ref|NM_004044.5 *Homo sapiens* 5-aminoimidazole-4-carboxamide ribonucleotide formyltransferase/IMP cyclohydrolase (ATIC), mRNA	3.17026
AB171508	gi|89276768|ref|NM_002747.3 *Homo sapiens* mitogen-activated protein kinase 4 (MAPK4), mRNA	3.12296
DC639656	gi|148596946|ref|NM_001098483.1 *Homo sapiens* chromosome 10 open reading frame 125 (C10orf125), transcript variant 1, mRNA	3.11887
AB048996	gi|211938419|ref|NM_002898.3 *Homo sapiens* RNA-binding motif, single stranded interacting protein 2 (RBMS2), mRNA	3.11444
DC630823	gi|215422360|ref|NM_004786.2 *Homo sapiens* thioredoxin-like 1 (TXNL1), transcript variant 1, mRNA	3.09601
AB173283	gi|197927150|ref|NM_006158.3 *Homo sapiens* neurofilament, light polypeptide (NEFL), mRNA	3.09117
DC633065	gi|34486089|ref|NM_004152.2 *Homo sapiens* ornithine decarboxylase antizyme 1 (OAZ1), mRNA	3.0599
AB174730	gi|115387109|ref|NM_017831.3 *Homo sapiens* ring finger protein 125 (RNF125), mRNA	3.03611
AB046044	gi|170650673|ref|NM_000440.2 *Homo sapiens* phosphodiesterase 6A, cGMP-specific, rod, alpha (PDE6A), mRNA	3.03568
AB179171	gi|82546851|ref|NM_175605.3 *Homo sapiens* intraflagellar transport 88 homolog (*Chlamydomonas*) (IFT88), transcript variant 1, mRNA	3.029
AB051155	gi|35493712|ref|NM_017890.3 *Homo sapiens* vacuolar protein sorting 13 homolog B (yeast) (VPS13B), transcript variant 5, mRNA	3.00764
AB072740	gi|155029549|ref|NM_178828.4 *Homo sapiens* chromosome 9 open reading frame 79 (C9orf79), mRNA	3.00753
BB881371	gi|162809333|ref|NM_002864.2 *Homo sapiens* pregnancy-zone protein (PZP), mRNA	2.99069
CJ463711	gi|215272411|ref|NM_001142334.1 *Homo sapiens* ataxin 2-binding protein 1 (A2BP1), transcript variant 6, mRNA	2.98758
AB170648	gi|5174424|ref|NM_006052.1 *Homo sapiens* Down syndrome critical region gene 3 (DSCR3), mRNA	2.9606
AB220465	gi|58331245|ref|NM_000817.2 *Homo sapiens* glutamate decarboxylase 1 (brain, 67 kDa) (GAD1), transcript variant GAD67, mRNA	2.95173
AB062990	gi|33149330|ref|NM_022463.3 *Homo sapiens* nucleoredoxin (NXN), mRNA	2.94787
AB220509	gi|139394620|ref|NM_006574.3 *Homo sapiens* chondroitin sulfate proteoglycan 5 (neuroglycan C) (CSPG5), mRNA	2.92569
AB070086	gi|22538813|ref|NM_002985.2 *Homo sapiens* chemokine (C-C motif) ligand 5 (CCL5), mRNA	2.92446
AB173147	gi|194294550|ref|NM_015989.4 *Homo sapiens* cysteine sulfinic acid decarboxylase (CSAD), mRNA	2.91509
AB051133	gi|28329444|ref|NM_014379.2 *Homo sapiens* potassium channel, subfamily V, member 1 (KCNV1), mRNA	2.87085
AB174705	gi|51477720|ref|NM_001003811.1 *Homo sapiens* testis-expressed 11 (TEX11), transcript variant 1, mRNA	2.86731
CJ469703	gi|169646771|ref|NM_002064.2 *Homo sapiens* glutaredoxin (thioltransferase) (GLRX), transcript variant 1, mRNA	2.85957
AB220438	gi|109633045|ref|NM_001042437.1 *Homo sapiens* ST3 beta-galactoside alpha-2,3-sialyltransferase 5 (ST3GAL5), transcript variant 2, mRNA	2.85364
BB881475	gi|170650673|ref|NM_000440.2 *Homo sapiens* phosphodiesterase 6A, cGMP-specific, rod, alpha (PDE6A), mRNA	2.84456
AB168610	gi|62632749|ref|NM_014616.1 *Homo sapiens* ATPase, class VI, type 11B (ATP11B), mRNA	2.84449
AB173806	gi|134031964|ref|NR_003491.1 *Homo sapiens* myocardial infarction associated transcript (nonprotein coding) (MIAT), noncoding RNA	2.82677
AB063045	gi|190570175|ref|NM_152906.4 *Homo sapiens* chromosome 22 open reading frame 25 (C22orf25), mRNA	2.80672
AB168446	gi|223278411|ref|NR_026782.1 *Homo sapiens* chromosome 1 open reading frame 175 (C1orf175), transcript variant 2, transcribed RNA	2.79215
CJ473171	gi|215490055|ref|NM_001142434.1 *Homo sapiens* meningioma-expressed antigen 5 (hyaluronidase) (MGEA5), transcript variant 2, mRNA	2.78342
CJ450383	gi|83641894|ref|NM_031157.2 *Homo sapiens* heterogeneous nuclear ribonucleoprotein A1 (HNRNPA1), transcript variant 2, mRNA	2.78321
DW528650	gi|58430810|ref|NM_148912.2 *Homo sapiens* abhydrolase domain-containing 11 (ABHD11), transcript variant 1, mRNA	2.77238
AB362499	gi|48255911|ref|NM_012338.3 *Homo sapiens* tetraspanin 12 (TSPAN12), mRNA	2.76878
AB173195	gi|196049386|ref|NM_002198.2 *Homo sapiens* interferon regulatory factor 1 (IRF1), mRNA	2.76544
AB168743	gi|188536107|ref|NM_001127458.1 *Homo sapiens* cardiolipin synthase 1 (CRLS1), transcript variant 2, mRNA	2.76042
CJ444006	gi|157151724|ref|NM_001004333.3 *Homo sapiens* ribonuclease, RNase K (RNASEK), mRNA	2.75719
DK583186	gi|156631002|ref|NM_006913.3 *Homo sapiens* ring finger protein 5 (RNF5), mRNA	2.75394
AB168582	gi|239757151|ref|XM_002345145.1 PREDICTED: *Homo sapiens* hypothetical protein LOC100292623 (LOC100292623), mRNA	2.75285
BB884235	gi|70906436|ref|NM_000509.4 *Homo sapiens* fibrinogen gamma chain (FGG), transcript variant gamma-A, mRNA	2.74831
AB070088	gi|148613875|ref|NM_144715.3 *Homo sapiens* EF-hand domain family, member B (EFHB), mRNA	2.74738
AB174502	gi|239757416|ref|XM_002345385.1 PREDICTED: *Homo sapiens* similar to hCG2019710 (LOC100294049), mRNA	2.73964
AB172306	gi|196162714|ref|NM_024786.2 *Homo sapiens* zinc finger, DHHC-type-containing 11 (ZDHHC11), mRNA	2.72049
BB878691	gi|19743563|ref|NM_000766.3 *Homo sapiens* cytochrome P450, family 2, subfamily A, polypeptide 13 (CYP2A13), mRNA	2.72031
AB174483	gi|55775474|ref|NM_194326.2 *Homo sapiens* ribosomal protein S19-binding protein 1 (RPS19BP1), mRNA	2.68579
DC632651	gi|22538474|ref|NM_018955.2 *Homo sapiens* ubiquitin B (UBB), mRNA	2.66418
AB168353	gi|197927266|ref|NM_004388.2 *Homo sapiens* chitobiase, di-N-acetyl (CTBS), mRNA	2.64444
AB169323	gi|156616291|ref|NM_018100.3 *Homo sapiens* EF-hand domain (C-terminal)-containing 1 (EFHC1), mRNA	2.6433
AB048961	gi|209413742|ref|NM_005458.6 *Homo sapiens* gamma-aminobutyric acid (GABA) B receptor, 2 (GABBR2), mRNA	2.63945
AB173162	gi|73747880|ref|NM_015113.3 *Homo sapiens* zinc finger, ZZ-type with EF-hand domain 1 (ZZEF1), mRNA	2.63631
AB179192	gi|237681201|ref|NM_019644.3 *Homo sapiens* ankyrin repeat domain 7 (ANKRD7), mRNA	2.62211
CJ469417	gi|169790802|ref|NM_005271.2 *Homo sapiens* glutamate dehydrogenase 1 (GLUD1), mRNA	2.62134
AB172772	gi|61835190|ref|NM_006578.3 *Homo sapiens* guanine nucleotide-binding protein (G protein), beta 5 (GNB5), transcript variant 1, mRNA	2.61592
DC857715	gi|169234652|ref|NM_007360.2 *Homo sapiens* killer cell lectin-like receptor subfamily K, member 1 (KLRK1), mRNA	2.6139
AB179131	gi|40807356|ref|NM_005094.2 *Homo sapiens* solute carrier family 27 (fatty acid transporter), member 4 (SLC27A4), mRNA	2.61058
DC647811	gi|92091576|ref|NM_015533.3 *Homo sapiens* dihydroxyacetone kinase 2 homolog (*S. cerevisiae*) (DAK), mRNA	2.60217
AB171456	gi|197927256|ref|NM_001134664.1 *Homo sapiens* sterile alpha motif domain-containing 13 (SAMD13), transcript variant 3, mRNA	2.59522
CJ490832	gi|169205007|ref|XM_001714899.1 PREDICTED: *Homo sapiens* hypothetical LOC100131988 (LOC100131988), mRNA	2.58063
DC635714	gi|57013275|ref|NM_006082.2 *Homo sapiens* tubulin, alpha 1b (TUBA1B), mRNA	2.57848
AB055358	gi|225735571|ref|NR_027416.1 *Homo sapiens* nuclear factor erythroid-derived 2-like 3 pseudogene (LOC100272146), noncoding RNA	2.57079
CJ458955	gi|197382308|ref|NM_183394.2 *Homo sapiens* Ca^++^-dependent secretion activator (CADPS), transcript variant 2, mRNA	2.55777
DC636940	gi|54112387|ref|NM_001005738.1 *Homo sapiens* formyl peptide receptor 2 (FPR2), transcript variant 2, mRNA	2.54881
CJ493104	gi|56676323|ref|NM_001001552.3 *Homo sapiens* LEM domain-containing 1 (LEMD1), mRNA	2.5487
AB172122	gi|239754513|ref|XM_001716238.2 PREDICTED: *Homo sapiens* hypothetical LOC100128081 (LOC100128081), mRNA	2.54837
CJ469678	gi|197304748|ref|NM_032727.3 *Homo sapiens* internexin neuronal intermediate filament protein, alpha (INA), mRNA	2.53491
AB070172	gi|32490571|ref|NM_012307.2 *Homo sapiens* erythrocyte membrane protein band 4.1-like 3 (EPB41L3), mRNA	2.52667
DW528013	gi|225735571|ref|NR_027416.1 *Homo sapiens* nuclear factor erythroid-derived 2-like 3 pseudogene (LOC100272146), noncoding RNA	2.52479
AB171587	gi|161169016|ref|NM_001111019.1 *Homo sapiens* neuron navigator 2 (NAV2), transcript variant 4, mRNA	2.5209
BB888693	gi|49574509|ref|NM_016013.2 *Homo sapiens* NADH dehydrogenase (ubiquinone) 1 alpha subcomplex, assembly factor 1 (NDUFAF1), mRNA	2.51576
BB897871	gi|207113180|ref|NM_001097577.2 *Homo sapiens* angiogenin, ribonuclease, RNase A family, 5 (ANG), transcript variant 2, mRNA	2.50309
AB168153	gi|222352148|ref|NM_018665.2 *Homo sapiens* DEAD (Asp-Glu-Ala-Asp) box polypeptide 43 (DDX43), mRNA	2.50236
AB173823	gi|148225856|ref|NM_001097579.1 *Homo sapiens* G protein-coupled receptor 34 (GPR34), transcript variant 4, mRNA	2.49912
AB168907	gi|194688136|ref|NM_002358.3 *Homo sapiens* MAD2 mitotic arrest deficient-like 1 (yeast) (MAD2L1), mRNA	2.49825
DK583616	gi|18390348|ref|NM_000972.2 *Homo sapiens* ribosomal protein L7a (RPL7A), mRNA	2.49444
CJ471599	gi|197245445|ref|NM_024958.2 *Homo sapiens* neurensin 2 (NRSN2), mRNA	2.49281
AB179111	gi|118572587|ref|NM_001761.2 *Homo sapiens* cyclin F (CCNF), mRNA	2.49071
AB060886	gi|23199979|ref|NM_022470.2 *Homo sapiens* zinc finger, matrin type 3 (ZMAT3), transcript variant 1, mRNA	2.48362
AB056810	gi|126131101|ref|NM_138694.3 *Homo sapiens* polycystic kidney and hepatic disease 1 (autosomal recessive) (PKHD1), transcript variant 1, mRNA	2.48299
DC645529	gi|4501988|ref|NM_001134.1 *Homo sapiens* alpha-fetoprotein (AFP), mRNA	2.46808
AB050420	gi|189095267|ref|NM_000554.4 *Homo sapiens* cone-rod homeobox (CRX), mRNA	2.45627
AB243403	gi|116235483|ref|NM_002701.4 *Homo sapiens* POU class 5 homeobox 1 (POU5F1), transcript variant 1, mRNA	2.4534
AB173020	gi|94420687|ref|NM_004233.3 *Homo sapiens* CD83 molecule (CD83), transcript variant 1, mRNA	2.44967
DC643036	gi|50409862|ref|NM_017584.5 *Homo sapiens* myoinositol oxygenase (MIOX), mRNA	2.44791
BB897024	gi|142976728|ref|NM_016245.3 *Homo sapiens* hydroxysteroid (17-beta) dehydrogenase 11 (HSD17B11), mRNA	2.43894
DW522619	gi|34147617|ref|NM_138807.2 *Homo sapiens* chromosome 3 open reading frame 31 (C3orf31), mRNA	2.43416
DC645828	gi|16332359|ref|NM_033487.1 *Homo sapiens* cell division cycle 2-like 1 (PITSLRE proteins) (CDC2L1), transcript variant 3, mRNA	2.43092
AB050260	gi|203098333|ref|NM_032133.4 *Homo sapiens* MYCBP-associated protein (MYCBPAP), mRNA	2.41286
CJ436262	gi|150010638|ref|NM_015276.1 *Homo sapiens* ubiquitin specific peptidase 22 (USP22), mRNA	2.39886
AB056381	gi|225735571|ref|NR_027416.1 *Homo sapiens* nuclear factor erythroid-derived 2-like 3 pseudogene (LOC100272146), noncoding RNA	2.39471
CJ443349	gi|83367079|ref|NM_003801.3 *Homo sapiens* glycosylphosphatidylinositol anchor attachment protein 1 homolog (yeast) (GPAA1), mRNA	2.39333
AB171767	gi|126273571|ref|NM_144586.5 *Homo sapiens* LY6/PLAUR domain-containing 1 (LYPD1), transcript variant 1, mRNA	2.393
AB056817	gi|58535452|ref|NM_001011649.1 *Homo sapiens* CDK5 regulatory subunit-associated protein 2 (CDK5RAP2), transcript variant 2, mRNA	2.39285
AB174345	gi|145208007|ref|NM_173688.2 *Homo sapiens* Na^+^/K^+^-transporting ATPase interacting 3 (NKAIN3), mRNA	2.39236
DC648733	gi|134133239|ref|NM_032151.4 *Homo sapiens* pterin-4 alpha-carbinolamine dehydratase/dimerization cofactor of hepatocyte nuclear factor 1 alpha (TCF1) 2 (PCBD2), mRNA	2.39231
CJ435057	gi|189083855|ref|NM_000815.4 *Homo sapiens* gamma-aminobutyric acid (GABA) A receptor, delta (GABRD), mRNA	2.38598
AB172049	gi|41281366|ref|NM_001440.2 *Homo sapiens* exostoses (multiple)-like 3 (EXTL3), mRNA	2.37312
CJ489397	gi|71143136|ref|NM_005342.2 *Homo sapiens* high-mobility group box 3 (HMGB3), mRNA	2.36471
AB170096	gi|42741653|ref|NM_007375.3 *Homo sapiens* TAR DNA-binding protein (TARDBP), mRNA	2.36217
AB056391	gi|169216999|ref|XM_001720515.1 PREDICTED: *Homo sapiens* similar to pro-pol protein (LOC100129323), mRNA	2.3496
AB071089	gi|167900475|ref|NM_001080850.2 *Homo sapiens* coiled-coil domain-containing 30 (CCDC30), mRNA	2.34558
DK579603	gi|84626579|ref|NM_025108.2 *Homo sapiens* chromosome 16 open reading frame 59 (C16orf59), mRNA	2.34371
CJ431113	gi|226246632|ref|NR_027451.1 *Homo sapiens* hypothetical LOC647979 (LOC647979), noncoding RNA	2.34265
DK580610	gi|34335291|ref|NM_003312.4 *Homo sapiens* thiosulfate sulfurtransferase (rhodanese) (TST), nuclear gene encoding mitochondrial protein, mRNA	2.3426
AB168450	gi|81295815|ref|NM_012337.2 *Homo sapiens* coiled-coil domain-containing 19 (CCDC19), mRNA	2.34063
DQ159931	gi|163659857|ref|NM_000828.4 *Homo sapiens* glutamate receptor, ionotrophic, AMPA 3 (GRIA3), transcript variant 2, mRNA	2.33939
AB173516	gi|36287116|ref|NM_014319.3 *Homo sapiens* LEM domain-containing 3 (LEMD3), mRNA	2.33925
AB173575	gi|56550100|ref|NM_020978.3 *Homo sapiens* amylase, alpha 2B (pancreatic) (AMY2B), mRNA	2.33548
AB169015	gi|93277104|ref|NM_173812.4 *Homo sapiens* dpy-19-like 2 (*C. elegans*) (DPY19L2), mRNA	2.33006
BB898675	gi|70906438|ref|NM_021870.2 *Homo sapiens* fibrinogen gamma chain (FGG), transcript variant gamma-B, mRNA	2.32385
DK579646	gi|153791317|ref|NM_032332.3 *Homo sapiens* mitogen-activated protein kinase organizer 1 (MORG1), transcript variant 2, mRNA	2.32308
AB071115	gi|111548669|ref|NM_153376.2 *Homo sapiens* coiled-coil domain-containing 96 (CCDC96), mRNA	2.31559
DC632824	gi|23110926|ref|NM_002799.2 *Homo sapiens* proteasome (prosome, macropain) subunit, beta type, 7 (PSMB7), mRNA	2.31501
BB896362	gi|188595719|ref|NM_005141.3 *Homo sapiens* fibrinogen beta chain (FGB), mRNA	2.3131
AB292416	gi|143770740|ref|NM_001083899.1 *Homo sapiens* glycoprotein VI (platelet) (GP6), transcript variant 1, mRNA	2.311
AB055350	gi|67782353|ref|NM_001024844.1 *Homo sapiens* CD82 molecule (CD82), transcript variant 2, mRNA	2.31014
AB168962	gi|210147405|ref|NM_152621.5 *Homo sapiens* sphingomyelin synthase 2 (SGMS2), transcript variant 1, mRNA	2.30806
AB168166	gi|156415985|ref|NM_014579.2 *Homo sapiens* solute carrier family 39 (zinc transporter), member 2 (SLC39A2), mRNA	2.30287
AB172981	gi|73695942|ref|NM_001010927.2 *Homo sapiens* T-cell lymphoma invasion and metastasis 2 (TIAM2), transcript variant 2, mRNA	2.29903
CJ441025	gi|153252025|ref|NM_001830.3 *Homo sapiens* chloride channel 4 (CLCN4), mRNA	2.29786
CJ445440	gi|42764686|ref|NM_022652.2 *Homo sapiens* dual specificity phosphatase 6 (DUSP6), transcript variant 2, mRNA	2.29536
AB179072	gi|156119614|ref|NM_006901.2 *Homo sapiens* myosin IXA (MYO9A), mRNA	2.28584
AB060229	gi|239756940|ref|XM_001718053.2 PREDICTED: *Homo sapiens* similar to CD300C antigen (LOC100130520), mRNA	2.28415
CJ480802	gi|71772428|ref|NM_001021.3 *Homo sapiens* ribosomal protein S17 (RPS17), mRNA	2.28212
DK581053	gi|63054873|ref|NM_001615.3 *Homo sapiens* actin, gamma 2, smooth muscle, enteric (ACTG2), mRNA	2.27782
AB046030	gi|169210010|ref|XR_040492.1 PREDICTED: *Homo sapiens* hypothetical LOC440386 (LOC440386), miscRNA	2.27371
AB174638	gi|44680147|ref|NM_203327.1 *Homo sapiens* solute carrier family 23 (nucleobase transporters), member 2 (SLC23A2), transcript variant 2, mRNA	2.2635
CJ469779	gi|95147340|ref|NM_004603.2 *Homo sapiens* syntaxin 1A (brain) (STX1A), mRNA	2.25959
DC632108	gi|225637497|ref|NR_003286.2 *Homo sapiens* 18S ribosomal RNA (LOC100008588), noncoding RNA	2.25815
AB170956	gi|65506441|ref|NM_000282.2 *Homo sapiens* propionyl coenzyme A carboxylase, alpha polypeptide (PCCA), nuclear gene encoding mitochondrial protein, transcript variant 1, mRNA	2.25785
DC858184	gi|13569959|ref|NM_030980.1 *Homo sapiens* interferon stimulated exonuclease gene 20 kDa-like 2 (ISG20L2), mRNA	2.25755
CJ470090	gi|169790838|ref|NM_004172.4 *Homo sapiens* solute carrier family 1 (glial high affinity glutamate transporter), member 3 (SLC1A3), mRNA	2.24996
DW522847	gi|215983055|ref|NM_031471.5 *Homo sapiens* fermitin family homolog 3 (*Drosophila*) (FERMT3), transcript variant URP2SF, mRNA	2.23483
DK577957	gi|186910295|ref|NM_001126102.1 *Homo sapiens* haptoglobin (HP), transcript variant 2, mRNA	2.23107
AB168531	gi|162951880|ref|NM_001112707.1 *Homo sapiens* tousled-like kinase 2 (TLK2), transcript variant B, mRNA	2.22872
AB047603	gi|149363673|ref|NM_012194.1 *Homo sapiens* chromosome 11 open reading frame 41 (C11orf41), mRNA	2.22681
DC642489	gi|66392201|ref|NM_002512.2 *Homo sapiens* nonmetastatic cells 2, protein (NM23B) expressed in (NME2), transcript variant 1, mRNA	2.22176
AB072760	gi|91754184|ref|NM_152763.3 *Homo sapiens* chromosome 1 open reading frame 62 (C1orf62), mRNA	2.2183
AB168708	gi|36287109|ref|NM_194429.1 *Homo sapiens* FGFR1 oncogene partner (FGFR1OP), transcript variant 2, mRNA	2.21393
AB168136	gi|93004101|ref|NM_005730.3 *Homo sapiens* CTD (carboxy-terminal domain, RNA polymerase II, polypeptide A) small phosphatase 2 (CTDSP2), mRNA	2.20887
DC641033	gi|108389126|ref|NM_001042353.1 *Homo sapiens* family with sequence similarity 110, member A (FAM110A), transcript variant 3, mRNA	2.20687
CJ453010	gi|19743893|ref|NM_133480.1 *Homo sapiens* transcriptional adaptor 3 (NGG1 homolog, yeast)-like (TADA3L), transcript variant 2, mRNA	2.20608
AB168419	gi|32996736|ref|NM_173083.2 *Homo sapiens* lin-9 homolog (C. elegans) (LIN9), mRNA	2.20292
AB169251	gi|148727318|ref|NM_001098529.1 *Homo sapiens* thioredoxin domain-containing 2 (spermatozoa) (TXNDC2), transcript variant 2, mRNA	2.2019
AB173492	gi|31542657|ref|NM_018099.3 *Homo sapiens* fatty acyl CoA reductase 2 (FAR2), mRNA	2.20171
CJ443285	gi|62955828|ref|NM_033428.1 *Homo sapiens* chromosome 9 open reading frame 123 (C9orf123), mRNA	2.19894
AB169808	gi|20336295|ref|NM_018380.2 *Homo sapiens* DEAD (Asp-Glu-Ala-Asp) box polypeptide 28 (DDX28), nuclear gene encoding mitochondrial protein, mRNA	2.1907
AB169213	gi|125625349|ref|NM_001790.3 *Homo sapiens* cell division cycle 25 homolog C (S. pombe) (CDC25C), transcript variant 1, mRNA	2.18403
CJ491693	gi|21361889|ref|NM_021633.2 *Homo sapiens* Kelch-like 12 (*Drosophila*) (KLHL12), mRNA	2.1801
BB876451	gi|109389366|ref|NM_000312.2 *Homo sapiens* protein C (inactivator of coagulation factors Va and VIIIa) (PROC), mRNA	2.17421
AB171871	gi|153070253|ref|NM_001099680.1 *Homo sapiens* MAGI family member, X-linked (MAGIX), transcript variant 2, mRNA	2.17287
AB173097	gi|198041927|ref|NM_139241.2 *Homo sapiens* FYVE, RhoGEF and PH domain-containing 4 (FGD4), mRNA	2.16791
AB046632	gi|136255215|ref|NM_207351.3 *Homo sapiens* proline-rich transmembrane protein 3 (PRRT3), mRNA	2.16598
DK577943	gi|84872083|ref|NR_002798.1 *Homo sapiens* napsin B aspartic peptidase pseudogene (NAPSB), noncoding RNA	2.16578
AB168792	gi|141802709|ref|NM_145263.2 *Homo sapiens* spermatogenesis associated 18 homolog (rat) (SPATA18), mRNA	2.16491
BB881148	gi|189458840|ref|NM_005942.3 *Homo sapiens* molybdenum cofactor synthesis 1 (MOCS1), transcript variant 2, mRNA	2.16393
CJ472360	gi|24497456|ref|NM_139136.2 *Homo sapiens* potassium voltage-gated channel, Shaw-related subfamily, member 2 (KCNC2), transcript variant 1, mRNA	2.15994
CJ430900	gi|94721262|ref|NM_001040446.1 *Homo sapiens* myotubularin-related protein 12 (MTMR12), mRNA	2.15957
AB171550	gi|19913413|ref|NM_014203.2 *Homo sapiens* adaptor-related protein complex 2, alpha 1 subunit (AP2A1), transcript variant 1, mRNA	2.1589
AB173954	gi|188595678|ref|NM_014959.2 *Homo sapiens* caspase recruitment domain family, member 8 (CARD8), mRNA	2.13719
AB071125	gi|89903024|ref|NM_001031735.2 *Homo sapiens* chromosome 19 open reading frame 36 (C19orf36), transcript variant 1, mRNA	2.13665
AB063014	gi|170650671|ref|NM_001122769.1 *Homo sapiens* Leber congenital amaurosis 5 (LCA5), transcript variant 2, mRNA	2.13524
DC631520	gi|189163527|ref|NM_001127700.1 *Homo sapiens* serpin peptidase inhibitor, clade A (alpha-1 antiproteinase, antitrypsin), member 1 (SERPINA1), transcript variant 4, mRNA	2.1306
AY742821	gi|59806358|ref|NM_006011.3 *Homo sapiens* ST8 alpha-N-acetyl-neuraminide alpha-2,8-sialyltransferase 2 (ST8SIA2), mRNA	2.12885
AK240628	gi|160298141|ref|NM_000668.4 *Homo sapiens* alcohol dehydrogenase 1B (class I), beta polypeptide (ADH1B), mRNA	2.12458
AB174195	gi|30794215|ref|NM_030961.1 *Homo sapiens* tripartite motif-containing 56 (TRIM56), mRNA	2.12446
DC646861	gi|91807120|ref|NM_033087.3 *Homo sapiens* asparagine-linked glycosylation 2, alpha-1,3-mannosyltransferase homolog (S. cerevisiae) (ALG2), transcript variant 1, mRNA	2.12291
AY650365	gi|27436932|ref|NM_172337.1 *Homo sapiens* orthodenticle homeobox 2 (OTX2), transcript variant 2, mRNA	2.11514
DW527197	gi|219555668|ref|NM_052855.3 *Homo sapiens* ankyrin repeat domain 40 (ANKRD40), mRNA	2.115
AB171287	gi|188497721|ref|NM_001127385.1 *Homo sapiens* cortexin 3 (CTXN3), transcript variant 2, mRNA	2.11438
AB173764	gi|219879811|ref|NM_005475.2 *Homo sapiens* SH2B adaptor protein 3 (SH2B3), mRNA	2.10791
DK582787	gi|221316657|ref|NM_004811.2 *Homo sapiens* leupaxin (LPXN), transcript variant 2, mRNA	2.10555
AB070128	gi|217416373|ref|NM_145038.2 *Homo sapiens* chromosome 2 open reading frame 39 (C2orf39), mRNA	2.10225
AB070165	gi|226491198|ref|NM_182496.2 *Homo sapiens* coiled-coil domain-containing 38 (CCDC38), mRNA	2.10075
DK577398	gi|52426772|ref|NM_002122.3 *Homo sapiens* major histocompatibility complex, class II, DQ alpha 1 (HLA-DQA1), mRNA	2.09004
AB169904	gi|34147601|ref|NM_004309.3 *Homo sapiens* rho GDP dissociation inhibitor (GDI) alpha (ARHGDIA), mRNA	2.08484
AB220503	gi|237681178|ref|NM_001160260.1 *Homo sapiens* cannabinoid receptor 1 (brain) (CNR1), transcript variant 6, mRNA	2.08434
AB173401	gi|239750034|ref|XR_039406.2 PREDICTED: *Homo sapiens* similar to yippee-like 5 (*Drosophila*) (LOC100132562), miscRNA	2.08407
AB171785	gi|253970447|ref|NM_014253.3 *Homo sapiens* odz, odd Oz/ten-m homolog 1(*Drosophila*) (ODZ1), transcript variant 3, mRNA	2.08184
AB171491	gi|117938287|ref|NM_004171.3 *Homo sapiens* solute carrier family 1 (glial high affinity glutamate transporter), member 2 (SLC1A2), mRNA	2.08087
AB174571	gi|182765446|ref|NM_001031711.2 *Homo sapiens* endoplasmic reticulum-Golgi intermediate compartment (ERGIC) 1 (ERGIC1), mRNA	2.07886
AB063092	gi|34577113|ref|NM_015576.1 *Homo sapiens* ELKS/RAB6-interacting/CAST family member 2 (ERC2), mRNA	2.07171
AB056378	gi|189163523|ref|NM_033064.4 *Homo sapiens* ataxia, cerebellar, Cayman type (ATCAY), mRNA	2.06758
AB055299	gi|163644324|ref|NM_001112732.1 *Homo sapiens* MCF.2 cell line derived transforming sequence-like (MCF2L), transcript variant 1, mRNA	2.06139
AB172748	gi|119220563|ref|NM_004852.2 *Homo sapiens* one cut homeobox 2 (ONECUT2), mRNA	2.05909
AB172478	gi|239746981|ref|XR_078603.1 PREDICTED: *Homo sapiens* similar to putative p150 (LOC100288106), miscRNA	2.05792
AB170807	gi|236459850|ref|NM_173569.3 *Homo sapiens* ubinuclein 2 (UBN2), mRNA	2.05471
EF208824	gi|239740919|ref|XM_002344047.1 PREDICTED: *Homo sapiens* similar to major histocompatibility complex, class II, DQ beta 1, transcript variant 2 (LOC100294318), mRNA	2.05031
AB169481	gi|150417992|ref|NM_033312.2 *Homo sapiens* CDC14 cell division cycle 14 homolog A (S. cerevisiae) (CDC14A), transcript variant 2, mRNA	2.04992
AB171520	gi|56243494|ref|NM_004586.2 *Homo sapiens* ribosomal protein S6 kinase, 90 kDa, polypeptide 3 (RPS6KA3), mRNA	2.0465
DC629151	gi|215982788|ref|NM_000477.5 *Homo sapiens* albumin (ALB), mRNA	2.04345
DC640591	gi|208609965|ref|NM_001135664.1 *Homo sapiens* RAB7, member RAS oncogene family-like 1 (RAB7L1), transcript variant 4, mRNA	2.04167
BB887273	gi|215,982,788|ref|NM_000477.5 *Homo sapiens* albumin (ALB), mRNA	2.0414
CJ435276	gi|75,812,975|ref|NM_001033574.1 *Homo sapiens* archaelysin family metallopeptidase 2 (AMZ2), transcript variant 6, mRNA	2.04064
DC643114	gi|33519462|ref|NM_004544.2 *Homo sapiens* NADH dehydrogenase (ubiquinone) 1 alpha subcomplex, 10, 42 kDa (NDUFA10), nuclear gene encoding mitochondrial protein, mRNA	2.03575
CJ436048	gi|62865867|ref|NM_004102.3 *Homo sapiens* fatty acid-binding protein 3, muscle and heart (mammary-derived growth inhibitor) (FABP3), mRNA	2.03528
AB179303	gi|196162694|ref|NM_003401.3 *Homo sapiens* X-ray repair complementing defective repair in Chinese hamster cells 4 (XRCC4), transcript variant 1, mRNA	2.03322
AB171313	gi|146219840|ref|NM_020709.1 *Homo sapiens* PNMA-like 2 (PNMAL2), mRNA	2.02925
AB173369	gi|149363694|ref|NM_001009984.1 *Homo sapiens* chromosome 20 open reading frame 194 (C20orf194), mRNA	2.01079
AB171481	gi|18496982|ref|NM_015526.1 *Homo sapiens* CAP-GLY domain-containing linker protein 3 (CLIP3), mRNA	2.00151
AB174068	gi|84872123|ref|NR_002833.1 *Homo sapiens* dpy-19-like 2 pseudogene 1 (*C. elegans*) (DPY19L2P1), noncoding RNA	1.99958
AB050434	gi|239753181|ref|XM_002345525.1 PREDICTED: *Homo sapiens* similar to hCG2041348 (LOC100293610), mRNA	1.99905
DK577438	gi|88999575|ref|NM_002622.4 *Homo sapiens* prefoldin subunit 1 (PFDN1), mRNA	1.99519
AB172315	gi|239753426|ref|XR_038411.2 PREDICTED: *Homo sapiens* similar to eukaryotic translation elongation factor 1 beta 2 (LOC646973), miscRNA	1.99372
BB895222	gi|38372939|ref|NM_001185.2 *Homo sapiens* alpha-2-glycoprotein 1, zinc-binding (AZGP1), mRNA	1.99214
AB173728	gi|111154086|ref|NM_020631.3 *Homo sapiens* pleckstrin homology domain-containing, family G (with RhoGef domain) member 5 (PLEKHG5), transcript variant 1, mRNA	1.98959
DC647709	gi|28416926|ref|NM_002560.2 *Homo sapiens* purinergic receptor P2X, ligand-gated ion channel, 4 (P2RX4), mRNA	1.98692
AB048919	gi|156766083|ref|NM_031418.2 *Homo sapiens* anoctamin 3 (ANO3), mRNA	1.98684
AB179103	gi|18860913|ref|NM_021818.2 *Homo sapiens* salvador homolog 1 (*Drosophila*) (SAV1), mRNA	1.97681
AB046073	gi|118498342|ref|NM_014861.2 *Homo sapiens* ATPase, Ca^++^ transporting, type 2C, member 2 (ATP2C2), mRNA	1.97604
AB172144	gi|190341103|ref|NM_015163.5 *Homo sapiens* tripartite motif-containing 9 (TRIM9), transcript variant 1, mRNA	1.97599
DK583369	gi|171460955|ref|NM_005800.4 *Homo sapiens* ubiquitin-specific peptidase like 1 (USPL1), mRNA	1.9752
AB174098	gi|110815799|ref|NM_024345.3 *Homo sapiens* DDB1 and CUL4-associated factor 10 (DCAF10), mRNA	1.97309
DW526268	gi|31083173|ref|NM_181078.1 *Homo sapiens* interleukin 21 receptor (IL21R), transcript variant 2, mRNA	1.97258
AB171701	gi|20544144|ref|NM_139062.1 *Homo sapiens* casein kinase 1, delta (CSNK1D), transcript variant 2, mRNA	1.96537
CJ493302	gi|17136150|ref|NM_004724.2 *Homo sapiens* ZW10, kinetochore associated, homolog (*Drosophila*) (ZW10), mRNA	1.96534
DK577545	gi|24797075|ref|NM_002121.4 *Homo sapiens* major histocompatibility complex, class II, DP beta 1 (HLA-DPB1), mRNA	1.96346
CJ458429	gi|71067335|ref|NM_031462.2 *Homo sapiens* CD99 molecule-like 2 (CD99L2), transcript variant 1, mRNA	1.96208
AB172752	gi|32698785|ref|NM_182490.1 *Homo sapiens* zinc finger protein 227 (ZNF227), mRNA	1.95671
AB171668	gi|40385866|ref|NM_199227.1 *Homo sapiens* methionine aminopeptidase 1D (MAP1D), mRNA	1.95399
AB051117	gi|82617625|ref|NM_001037293.1 *Homo sapiens* paralemmin 2 (PALM2), transcript variant 2, mRNA	1.9528
AB169059	gi|91992151|ref|NM_000616.3 *Homo sapiens* CD4 molecule (CD4), mRNA	1.95226
CJ443230	gi|46094085|ref|NM_022758.4 *Homo sapiens* chromosome 6 open reading frame 106 (C6orf106), transcript variant 2, mRNA	1.95082
AB178987	gi|38045951|ref|NM_021030.2 *Homo sapiens* zinc finger protein 14 (ZNF14), mRNA	1.94816
AB172387	gi|163659919|ref|NM_052839.3 *Homo sapiens* pannexin 2 (PANX2), transcript variant 1, mRNA	1.94536
AB168775	gi|223468671|ref|NM_001145135.1 *Homo sapiens* carnitine palmitoyltransferase 1B (muscle) (CPT1B), nuclear gene encoding mitochondrial protein, transcript variant 6, mRNA	1.94062
DC633198	gi|206725531|ref|NM_001826.2 *Homo sapiens* CDC28 protein kinase regulatory subunit 1B (CKS1B), transcript variant 1, mRNA	1.93773
AB172044	gi|25168266|ref|NM_170709.1 *Homo sapiens* serum/glucocorticoid-regulated kinase family, member 3 (SGK3), transcript variant 2, mRNA	1.93543
DK578185	gi|239754745|ref|XM_002346052.1 PREDICTED: *Homo sapiens* hypothetical protein LOC100293771 (LOC100293771), mRNA	1.93421
AB171597	gi|113951732|ref|NM_012095.4 *Homo sapiens* adaptor-related protein complex 3, mu 1 subunit (AP3M1), transcript variant 2, mRNA	1.93313
AB179405	gi|31543301|ref|NM_032600.2 *Homo sapiens* coiled-coil domain-containing 54 (CCDC54), mRNA	1.93038
AB179267	gi|37594443|ref|NM_015896.2 *Homo sapiens* zinc finger, MYND-type-containing 10 (ZMYND10), mRNA	1.92859
DC640525	gi|35493837|ref|NM_004902.2 *Homo sapiens* RNA-binding motif protein 39 (RBM39), transcript variant 2, mRNA	1.92337
AB049869	gi|239753181|ref|XM_002345525.1 PREDICTED: *Homo sapiens* similar to hCG2041348 (LOC100293610), mRNA	1.92159
DW528250	gi|52487034|ref|NM_004618.3 *Homo sapiens* topoisomerase (DNA) III alpha (TOP3A), mRNA	1.91924
DC636463	gi|78214521|ref|NM_001035258.1 *Homo sapiens* ribosomal protein L38 (RPL38), transcript variant 2, mRNA	1.91826
AB179052	gi|115511031|ref|NM_004432.2 *Homo sapiens* ELAV- (embryonic lethal, abnormal vision, *Drosophila*-) like 2 (Hu antigen B) (ELAVL2), mRNA	1.91696
AB168809	gi|37622352|ref|NM_003551.2 *Homo sapiens* nonmetastatic cells 5, protein expressed in nucleoside-diphosphate kinase (NME5), mRNA	1.91336
CJ435007	gi|115527063|ref|NM_004859.3 *Homo sapiens* clathrin, heavy chain (Hc) (CLTC), mRNA	1.91318
AB171499	gi|50845406|ref|NM_031444.2 *Homo sapiens* chromosome 22 open reading frame 13 (C22orf13), mRNA	1.91088
DC647333	gi|118600974|ref|NM_007269.2 *Homo sapiens* syntaxin-binding protein 3 (STXBP3), mRNA	1.90575
AB172403	gi|142976637|ref|NM_017420.3 *Homo sapiens* SIX homeobox 4 (SIX4), mRNA	1.89362
AB174282	gi|31543080|ref|NM_016210.2 *Homo sapiens* chromosome 3 open reading frame 18 (C3orf18), mRNA	1.89271
DC648759	gi|6382072|ref|NM_005258.2 *Homo sapiens* GTP cyclohydrolase I feedback regulator (GCHFR), mRNA	1.88951
AB169033	gi|195927038|ref|NM_001786.3 *Homo sapiens* cell division cycle 2, G1 to S and G2 to M (CDC2), transcript variant 1, mRNA	1.88515
AB173309	gi|209447072|ref|NM_001135806.1 *Homo sapiens* synaptotagmin I (SYT1), transcript variant 3, mRNA	1.88194
AB063003	gi|116063563|ref|NM_018218.2 *Homo sapiens* ubiquitin-specific peptidase 40 (USP40), mRNA	1.88102
AB171041	gi|86787650|ref|NM_014800.9 *Homo sapiens* engulfment and cell motility 1 (ELMO1), transcript variant 1, mRNA	1.87945
CJ470094	gi|209364624|ref|NM_001822.4 *Homo sapiens* chimerin (chimaerin) 1 (CHN1), transcript variant 1, mRNA	1.87132
AB171236	gi|19743893|ref|NM_133480.1 *Homo sapiens* transcriptional adaptor 3 (NGG1 homolog, yeast)-like (TADA3L), transcript variant 2, mRNA	1.86897
BB885210	gi|32484974|ref|NM_006721.2 *Homo sapiens* adenosine kinase (ADK), transcript variant ADK-long, mRNA	1.86865
AB169067	gi|188528615|ref|NM_182911.3 *Homo sapiens* testis-specific, 10 (TSGA10), transcript variant 2, mRNA	1.86706
CJ464698	gi|221307560|ref|NR_026669.1 *Homo sapiens* synaptosomal-associated protein, 91 kDa homolog (mouse) (SNAP91), transcript variant 2, transcribed RNA	1.86542
AB179482	gi|51173716|ref|NM_006720.3 *Homo sapiens* actin-binding LIM protein 1 (ABLIM1), transcript variant 4, mRNA	1.85972
CJ442615	gi|239745120|ref|XR_015162.2 PREDICTED: *Homo sapiens* hypothetical protein LOC727880 (LOC727880), miscRNA	1.85021
CJ435208	gi|170650722|ref|NM_014236.3 *Homo sapiens* glyceronephosphate O-acyltransferase (GNPAT), mRNA	1.8499
AY650307	gi|51599155|ref|NM_001273.2 *Homo sapiens* chromodomain helicase DNA-binding protein 4 (CHD4), mRNA	1.84509
DW525872	gi|77404354|ref|NM_003908.3 *Homo sapiens* eukaryotic translation initiation factor 2, subunit 2 beta, 38 kDa (EIF2S2), mRNA	1.84501
DW529999	gi|78190459|ref|NM_000978.3 *Homo sapiens* ribosomal protein L23 (RPL23), mRNA	1.84409
AB174451	gi|223941821|ref|NM_014342.3 *Homo sapiens* mitochondrial carrier homolog 2 (*C. elegans*) (MTCH2), nuclear gene encoding mitochondrial protein, mRNA	1.84017
AB066534	gi|188528627|ref|NM_033109.3 *Homo sapiens* polyribonucleotide nucleotidyltransferase 1 (PNPT1), mRNA	1.83655
BB888999	gi|160298191|ref|NM_000507.3 *Homo sapiens* fructose-1,6-bisphosphatase 1 (FBP1), transcript variant 1, mRNA	1.8352
DC637318	gi|209977038|ref|NM_016074.3 *Homo sapiens* bolA homolog 1 (*E. coli*) (BOLA1), mRNA	1.83477
AB169205	gi|109948303|ref|NM_018225.2 *Homo sapiens* smu-1 suppressor of mec-8 and unc-52 homolog (*C. elegans*) (SMU1), mRNA	1.82627
CJ441961	gi|19913444|ref|NM_016257.2 *Homo sapiens* hippocalcin-like 4 (HPCAL4), mRNA	1.8159
AY650384	gi|141803509|ref|NM_058164.2 *Homo sapiens* olfactomedin 2 (OLFM2), mRNA	1.81587
DC647305	gi|38372918|ref|NM_001728.2 *Homo sapiens* basigin (Ok blood group) (BSG), transcript variant 1, mRNA	1.8093
AB172260	gi|112382251|ref|NM_178313.2 *Homo sapiens* spectrin, beta, nonerythrocytic 1 (SPTBN1), transcript variant 2, mRNA	1.80742
AB173850	gi|194097340|ref|NM_002616.2 *Homo sapiens* period homolog 1 (*Drosophila*) (PER1), mRNA	1.80415
AB168762	gi|242247096|ref|NM_001340.3 *Homo sapiens* cylicin, basic protein of sperm head cytoskeleton 2 (CYLC2), mRNA	1.80079
AB173856	gi|60302919|ref|NM_001752.2 *Homo sapiens* catalase (CAT), mRNA	1.79676
AB060862	gi|221219051|ref|NM_031924.4 *Homo sapiens* radial spoke 3 homolog (*Chlamydomonas*) (RSPH3), mRNA	1.79563
CJ470793	gi|224586819|ref|NR_027265.1 *Homo sapiens* Golgi apparatus protein 1 (GLG1), transcript variant 5, transcribed RNA	1.79405
DW528583	gi|239787833|ref|NM_015139.2 *Homo sapiens* solute carrier family 35 (UDP-glucuronic acid/UDP-N-acetylgalactosamine dual transporter), member D1 (SLC35D1), mRNA	1.79359
DK580881	gi|194394144|ref|NM_145870.2 *Homo sapiens* glutathione transferase zeta 1 (GSTZ1), transcript variant 1, mRNA	1.792
AB173997	gi|225543100|ref|NR_027378.1 *Homo sapiens* hypothetical LOC643763 (LOC643763), noncoding RNA	1.79131
AY650356	gi|223718142|ref|NM_173054.2 *Homo sapiens* reelin (RELN), transcript variant 2, mRNA	1.78729
DK584117	gi|15967154|ref|NM_016558.2 *Homo sapiens* SCAN domain-containing 1 (SCAND1), transcript variant 1, mRNA	1.78008
DC621384	gi|15431296|ref|NM_000977.2 *Homo sapiens* ribosomal protein L13 (RPL13), transcript variant 1, mRNA	1.77763
DK577712	gi|109148541|ref|NM_001605.2 *Homo sapiens* alanyl-tRNA synthetase (AARS), mRNA	1.77723
AB174251	gi|253314435|ref|NR_027995.1 *Homo sapiens* ankyrin repeat domain 20 family, member A2 pseudogene (LOC284232), noncoding RNA	1.77027
AB174247	gi|50897295|ref|NM_001002923.1 *Homo sapiens* IGF-like family member 4 (IGFL4), mRNA	1.76977
CJ490195	gi|78190459|ref|NM_000978.3 *Homo sapiens* ribosomal protein L23 (RPL23), mRNA	1.76786
AB171831	gi|167466275|ref|NM_152542.3 *Homo sapiens* protein phosphatase 1K (PP2C domain containing) (PPM1K), mRNA	1.76709
DK582810	gi|90652856|ref|NM_032818.2 *Homo sapiens* chromosome 9 open reading frame 100 (C9orf100), mRNA	1.765
AB170534	gi|108773786|ref|NM_000321.2 *Homo sapiens* retinoblastoma 1 (RB1), mRNA	1.76182
AB171096	gi|110347436|ref|NM_001042545.1 *Homo sapiens* latent transforming growth factor beta-binding protein 4 (LTBP4), transcript variant 3, mRNA	1.75594
AB168611	gi|21071068|ref|NM_004865.2 *Homo sapiens* TBP-like 1 (TBPL1), mRNA	1.74839
CJ492188	gi|30181234|ref|NM_003447.2 *Homo sapiens* zinc finger protein 165 (ZNF165), mRNA	1.74573
AB171700	gi|115527063|ref|NM_004859.3 *Homo sapiens* clathrin, heavy chain (Hc) (CLTC), mRNA	1.74566
AB171366	gi|22748942|ref|NM_152445.1 *Homo sapiens* family with sequence similarity 161, member B (FAM161B), mRNA	1.74405
AB168566	gi|148664196|ref|NM_017950.2 *Homo sapiens* coiled-coil domain-containing 40 (CCDC40), mRNA	1.74135
AB171657	gi|221316692|ref|NM_198449.2 *Homo sapiens* embigin homolog (mouse) (EMB), mRNA	1.73933
AB056808	gi|71772767|ref|NM_152826.2 *Homo sapiens*-sorting nexin 1 (SNX1), transcript variant 3, mRNA	1.73686
AB168849	gi|156766042|ref|NM_001103146.1 *Homo sapiens* GRB10-interacting GYF protein 2 (GIGYF2), transcript variant 3, mRNA	1.73412
AB172848	gi|95113665|ref|NM_018157.2 *Homo sapiens* resistance to inhibitors of cholinesterase 8 homolog B (*C. elegans*) (RIC8B), mRNA	1.72749
AB048894	gi|148727250|ref|NM_007137.2 *Homo sapiens* zinc finger protein 81 (ZNF81), mRNA	1.71845
DW524469	gi|239753181|ref|XM_002345525.1 PREDICTED: *Homo sapiens* similar to hCG2041348 (LOC100293610), mRNA	1.71582
AB173566	gi|89242130|ref|NM_014305.2 *Homo sapiens* TDP-glucose 4,6-dehydratase (TGDS), mRNA	1.71406
DC634783	gi|116812576|ref|NM_016019.2 *Homo sapiens* LUC7-like 2 (*S. cerevisiae*) (LUC7L2), mRNA	1.71163
AB168438	gi|64276485|ref|NM_005869.2 *Homo sapiens* serologically defined colon cancer antigen 10 (SDCCAG10), mRNA	1.71108
AB174725	gi|169194555|ref|XR_040716.1 PREDICTED: *Homo sapiens* hypothetical LOC439950 (LOC439950), miscRNA	1.70933
AB170786	gi|208879448|ref|NM_006265.2 *Homo sapiens* RAD21 homolog (*S. pombe*) (RAD21), mRNA	1.70551
CJ431422	gi|117938253|ref|NM_001077441.1 *Homo sapiens* BCL2-associated transcription factor 1 (BCLAF1), transcript variant 3, mRNA	1.70446
AB048954	gi|148596971|ref|NM_014951.2 *Homo sapiens* zinc finger protein 365 (ZNF365), transcript variant A, mRNA	1.70334
AB173447	gi|40288292|ref|NM_000361.2 *Homo sapiens* thrombomodulin (THBD), mRNA	1.70293
AB173287	gi|242117988|ref|NM_014702.4 *Homo sapiens* KIAA0408 (KIAA0408), mRNA	1.70162
CJ489820	gi|218505834|ref|NM_001142782.1 *Homo sapiens* membrane-associated guanylate kinase, WW, and PDZ domain-containing 3 (MAGI3), transcript variant 1, mRNA	1.68381
AB173372	gi|78190481|ref|NM_025221.5 *Homo sapiens* Kv channel-interacting protein 4 (KCNIP4), transcript variant 1, mRNA	1.68058
AB172865	gi|31795545|ref|NM_012450.2 *Homo sapiens* solute carrier family 13 (sodium/sulfate symporters), member 4 (SLC13A4), mRNA	1.67878
AB168329	gi|223468562|ref|NM_005628.2 *Homo sapiens* solute carrier family 1 (neutral amino acid transporter), member 5 (SLC1A5), transcript variant 1, mRNA	1.67642
AB171546	gi|55956903|ref|NM_005922.2 *Homo sapiens* mitogen-activated protein kinase kinase kinase 4 (MAP3K4), transcript variant 1, mRNA	1.67151
AB063093	gi|194248055|ref|NM_002045.3 *Homo sapiens* growth-associated protein 43 (GAP43), transcript variant 2, mRNA	1.66805
AB220449	gi|23510394|ref|NM_138966.2 *Homo sapiens* neuropilin- (NRP-) and tolloid- (TLL-) like 1 (NETO1), transcript variant 3, mRNA	1.66789
AB169208	gi|22547155|ref|NM_002018.2 *Homo sapiens* flightless I homolog (*Drosophila*) (FLII), mRNA	1.66361
AB168324	gi|116014337|ref|NM_030981.2 *Homo sapiens* RAB1B, member RAS oncogene family (RAB1B), mRNA	1.66298
AB169835	gi|50726964|ref|NM_013392.2 *Homo sapiens* nuclear receptor-binding protein 1 (NRBP1), mRNA	1.65785
AB173501	gi|195539333|ref|NM_018176.3 *Homo sapiens* leucine-rich repeat LGI family, member 2 (LGI2), mRNA	1.6574
DC630946	gi|183227689|ref|NM_002049.3 *Homo sapiens* GATA-binding protein 1 (globin transcription factor 1) (GATA1), mRNA	1.65657
AB063075	gi|239743824|ref|XM_001128647.3 PREDICTED: *Homo sapiens* hypothetical LOC728701 (LOC728701), mRNA	1.65564
AB169782	gi|38261964|ref|NM_198399.1 *Homo sapiens* cyclic AMP-regulated phosphoprotein, 21 kD (ARPP-21), transcript variant 2, mRNA	1.65067
CJ477467	gi|133778911|ref|NM_003309.2 *Homo sapiens* TSPY-like 1 (TSPYL1), mRNA	1.65062
BB900725	gi|31542685|ref|NM_025125.2 *Homo sapiens* chromosome 10 open reading frame 57 (C10orf57), mRNA	1.64409
AB220555	gi|208973250|ref|NM_003702.3 *Homo sapiens* regulator of G-protein signaling 20 (RGS20), transcript variant 2, mRNA	1.64376
AB171804	gi|66932910|ref|NM_014676.2 *Homo sapiens* pumilio homolog 1 (*Drosophila*) (PUM1), transcript variant 2, mRNA	1.63724
DC625559	gi|4557320|ref|NM_000039.1 *Homo sapiens* apolipoprotein A-I (APOA1), mRNA	1.631
AB172266	gi|170932491|ref|NM_030770.2 *Homo sapiens* transmembrane protease, serine 5 (TMPRSS5), mRNA	1.62995
AB173763	gi|62953115|ref|NM_001017523.1 *Homo sapiens* BTB (POZ) domain-containing 11 (BTBD11), transcript variant b, mRNA	1.62499
AB172974	gi|111161293|ref|NM_005746.2 *Homo sapiens* nicotinamide phosphoribosyltransferase (NAMPT), mRNA	1.62078
AB179155	gi|187608347|ref|NM_145046.3 *Homo sapiens* calreticulin 3 (CALR3), mRNA	1.6111
AB169148	gi|153792481|ref|NM_033048.4 *Homo sapiens* CPX chromosome region, candidate 1 (CPXCR1), mRNA	1.60985
AB171264	gi|130977817|ref|NM_024549.4 *Homo sapiens* tectonic family member 1 (TCTN1), transcript variant 3, mRNA	1.60704
AB172446	gi|193083128|ref|NM_001128920.1 *Homo sapiens* MAP/microtubule affinity-regulating kinase 3 (MARK3), transcript variant 4, mRNA	1.60471
DC852298	gi|195972796|ref|NM_001130917.1 *Homo sapiens* leukocyte immunoglobulin-like receptor, subfamily A (with TM domain), member 2 (LILRA2), transcript variant 1, mRNA	1.59852
AB046637	gi|209571546|ref|NM_018095.4 *Homo sapiens* Kelch repeat and BTB (POZ) domain-containing 4 (KBTBD4), transcript variant 1, mRNA	1.58107
CJ445723	gi|90903230|ref|NM_002111.6 *Homo sapiens* huntingtin (HTT), mRNA	1.57689
DC630899	gi|83641894|ref|NM_031157.2 *Homo sapiens* heterogeneous nuclear ribonucleoprotein A1 (HNRNPA1), transcript variant 2, mRNA	1.5766
AB168476	gi|219555742|ref|NM_015335.3 *Homo sapiens* mediator complex subunit 13-like (MED13L), mRNA	1.57355
DC642541	gi|20357546|ref|NM_004231.2 *Homo sapiens* ATPase, H+ transporting, lysosomal 14 kDa, V1 subunit F (ATP6V1F), mRNA	1.57215
AB170370	gi|224177554|ref|NM_002340.5 *Homo sapiens* lanosterol synthase (2,3-oxidosqualene-lanosterol cyclase) (LSS), transcript variant 1, mRNA	1.57006
DC636538	gi|71164876|ref|NM_001014.3 *Homo sapiens* ribosomal protein S10 (RPS10), mRNA	1.56413
DC648258	gi|4557818|ref|NM_000277.1 *Homo sapiens* phenylalanine hydroxylase (PAH), mRNA	1.56396
AB168688	gi|75709218|ref|NM_001324.2 *Homo sapiens* cleavage stimulation factor, 3′ pre-RNA, subunit 1, 50 kDa (CSTF1), transcript variant 2, mRNA	1.56349
CJ486539	gi|194018543|ref|NM_031451.4 *Homo sapiens* testis expressed 101 (TEX101), transcript variant 1, mRNA	1.55338
AB173591	gi|56699472|ref|NM_006298.2 *Homo sapiens* zinc finger protein 192 (ZNF192), mRNA	1.54892
AB168460	gi|56090619|ref|NM_001007531.1 *Homo sapiens* NFKB-activating protein-like (NKAPL), mRNA	1.54807
AB046102	gi|215272394|ref|NM_001080475.2 *Homo sapiens* pleckstrin homology domain containing, family M, member 3 (PLEKHM3), mRNA	1.53926
AB097526	gi|46409303|ref|NM_207332.1 *Homo sapiens* glutamate-rich 1 (ERICH1), mRNA	1.53642
AB052134	gi|227430412|ref|NM_024827.3 *Homo sapiens* histone deacetylase 11 (HDAC11), transcript variant 1, mRNA	1.53543
AB170181	gi|21265090|ref|NM_007208.2 *Homo sapiens* mitochondrial ribosomal protein L3 (MRPL3), nuclear gene encoding mitochondrial protein, mRNA	1.52903
AB171241	gi|116235443|ref|NM_138421.2 *Homo sapiens* serum amyloid A-like 1 (SAAL1), mRNA	1.52713
AB171237	gi|48675815|ref|NM_015723.2 *Homo sapiens* patatin-like phospholipase domain-containing 8 (PNPLA8), mRNA	1.52587
DC625517	gi|47578120|ref|NM_177947.2 *Homo sapiens* armadillo repeat containing, X-linked 3 (ARMCX3), transcript variant 2, mRNA	1.52547
AB168964	gi|87159814|ref|NM_001696.3 *Homo sapiens* ATPase, H+ transporting, lysosomal 31 kDa, V1 subunit E1 (ATP6V1E1), transcript variant 1, mRNA	1.52424
DC631115	gi|239752151|ref|XM_002348112.1 PREDICTED: *Homo sapiens* similar to immunoglobulin lambda locus (LOC100290481), mRNA	1.51711
DC640134	gi|208609986|ref|NM_014655.2 *Homo sapiens* solute carrier family 25, member 44 (SLC25A44), transcript variant 1, mRNA	1.51672
AB173691	gi|94536855|ref|NM_013301.2 *Homo sapiens* coiled-coil domain-containing 106 (CCDC106), mRNA	1.50477
AB168370	gi|154744869|ref|NM_022752.5 *Homo sapiens* zinc finger protein 574 (ZNF574), mRNA	1.50452

**Table 2:** Genes that are downregulated in TM cells.

Accession number	Human RefSeq description	Fold change
DC624859	gi|215982788|ref|NM_000477.5 *Homo sapiens* albumin (ALB), mRNA	0.11952
AB171761	gi|148271103|ref|NM_173495.2 *Homo sapiens* patched domain-containing 1 (PTCHD1), mRNA	0.13543
AB047615	gi|70780382|ref|NM_004285.3 *Homo sapiens* hexose-6-phosphate dehydrogenase (glucose 1-dehydrogenase) (H6PD), mRNA	0.17649
DC621007	gi|38016905|ref|NR_001578.1 *Homo sapiens* L-threonine dehydrogenase (TDH), noncoding RNA	0.20061
CJ443677	gi|38327038|ref|NM_002154.3 *Homo sapiens* heat shock 70 kDa protein 4 (HSPA4), mRNA	0.2086
DC622138	gi|145386530|ref|NM_001084392.1 *Homo sapiens* D-dopachrome tautomerase (DDT), transcript variant 2, mRNA	0.21345
BB891761	gi|33519462|ref|NM_004544.2 *Homo sapiens* NADH dehydrogenase (ubiquinone) 1 alpha subcomplex, 10, 42 kDa (NDUFA10), nuclear gene encoding mitochondrial protein, mRNA	0.23147
CJ444181	gi|226437566|ref|NM_001018060.2 *Homo sapiens* apoptosis-inducing factor, mitochondrion-associated 3 (AIFM3), nuclear gene encoding mitochondrial protein, transcript variant 2, mRNA	0.24045
AB171890	gi|118572602|ref|NM_001079514.1 *Homo sapiens* ubinuclein 1 (UBN1), transcript variant 2, mRNA	0.24676
AB174511	gi|153792041|ref|NM_020823.1 *Homo sapiens* transmembrane protein 181 (TMEM181), mRNA	0.29472
AB168319	gi|116256484|ref|NM_006781.3 *Homo sapiens* chromosome 6 open reading frame 10 (C6orf10), mRNA	0.3105
DW526909	gi|20302159|ref|NM_005999.2 *Homo sapiens* translin-associated factor X (TSNAX), mRNA	0.32949
AB173471	gi|154354995|ref|NM_002222.4 *Homo sapiens* inositol 1,4,5-triphosphate receptor, type 1 (ITPR1), transcript variant 2, mRNA	0.33892
BB898986	gi|167003944|ref|NM_000204.3 *Homo sapiens* complement factor I (CFI), mRNA	0.34234
DK578390	gi|56788350|ref|NM_001008695.1 *Homo sapiens* THAP domain-containing 7 (THAP7), transcript variant 2, mRNA	0.35502
CJ444326	gi|209413724|ref|NM_003692.3 *Homo sapiens* transmembrane protein with EGF-like and two follistatin-like domains 1 (TMEFF1), mRNA	0.3719
AB048874	gi|239753181|ref|XM_002345525.1 PREDICTED: *Homo sapiens* similar to hCG2041348 (LOC100293610), mRNA	0.38524
DC635743	gi|239750740|ref|XM_002347480.1 PREDICTED: *Homo sapiens* similar to hCG2038941 (LOC100290006), mRNA	0.3882
CJ442045	gi|96975096|ref|NM_016577.3 *Homo sapiens* RAB6B, member RAS oncogene family (RAB6B), mRNA	0.39034
BB897881	gi|31542685|ref|NM_025125.2 *Homo sapiens* chromosome 10 open reading frame 57 (C10orf57), mRNA	0.39308
AB168422	gi|194306536|ref|NM_144594.2 *Homo sapiens* gametocyte-specific factor 1 (GTSF1), mRNA	0.43061
DW524779	gi|226342870|ref|NR_027449.1 *Homo sapiens* TBC1 domain family, member 15 (TBC1D15), transcript variant 4, transcribed RNA	0.44075
DC630545	gi|39812105|ref|NM_198941.1 *Homo sapiens* serine incorporator 3 (SERINC3), transcript variant 2, mRNA	0.44508
AB172901	gi|42544225|ref|NM_020857.2 *Homo sapiens* vacuolar protein sorting 18 homolog (*S. cerevisiae*) (VPS18), mRNA	0.44872
DW528888	gi|40068463|ref|NM_020732.2 *Homo sapiens* AT-rich interactive domain 1B (SWI1-like) (ARID1B), transcript variant 2, mRNA	0.44985
DC636880	gi|17738314|ref|NM_006835.2 *Homo sapiens* cyclin I (CCNI), mRNA	0.45196
AB220379	gi|185134767|ref|NM_002524.3 *Homo sapiens* neuroblastoma RAS viral (v-ras) oncogene homolog (NRAS), mRNA	0.46121
AB055316	gi|226437631|ref|NM_001004339.2 *Homo sapiens* zyg-11 homolog A (*C. elegans*) (ZYG11A), mRNA	0.46399
DC641070	gi|58331227|ref|NM_005223.3 *Homo sapiens* deoxyribonuclease I (DNASE1), mRNA	0.46575
AB056428	gi|49574533|ref|NM_032782.3 *Homo sapiens* hepatitis A virus cellular receptor 2 (HAVCR2), mRNA	0.46705
AB168577	gi|146260272|ref|NM_001085451.1 *Homo sapiens* leukemia NUP98 fusion partner 1 (LNP1), mRNA	0.46891
AB048999	gi|225735571|ref|NR_027416.1 *Homo sapiens* nuclear factor erythroid-derived 2-like 3 pseudogene (LOC100272146), noncoding RNA	0.47824
AB174085	gi|142360382|ref|NM_176815.3 *Homo sapiens* dihydrofolate reductase-like 1 (DHFRL1), mRNA	0.49049
DC642335	gi|148491081|ref|NM_001343.2 *Homo sapiens*-disabled homolog 2, mitogen-responsive phosphoprotein (*Drosophila*) (DAB2), mRNA	0.4971
AB173771	gi|38176290|ref|NM_001233.3 *Homo sapiens* caveolin 2 (CAV2), transcript variant 1, mRNA	0.49863
DW523198	gi|145312264|ref|NM_033266.3 *Homo sapiens* endoplasmic reticulum to nucleus signaling 2 (ERN2), mRNA	0.50158
AF492282	gi|52630343|ref|NM_021983.4 *Homo sapiens* major histocompatibility complex, class II, DR beta 4 (HLA-DRB4), mRNA	0.50533
AB047937	gi|194097480|ref|NM_020412.4 *Homo sapiens* chromatin-modifying protein 1B (CHMP1B), mRNA	0.51198
AB179165	gi|118136291|ref|NM_006465.2 *Homo sapiens* AT-rich interactive domain 3B (bright-like) (ARID3B), mRNA	0.51349
AJ585530	gi|75709168|ref|NM_002260.3 *Homo sapiens* killer cell lectin-like receptor subfamily C, member 2 (KLRC2), mRNA	0.52335
AB170944	gi|40255250|ref|NM_144635.3 *Homo sapiens* family with sequence similarity 131, member A (FAM131A), mRNA	0.55244
AB171281	gi|154759258|ref|NM_003127.2 *Homo sapiens* spectrin, alpha, nonerythrocytic 1 (alpha-fodrin) (SPTAN1), transcript variant 2, mRNA	0.555
AB172429	gi|224994204|ref|NM_001145853.1 *Homo sapiens* Wolfram syndrome 1 (wolframin) (WFS1), transcript variant 2, mRNA	0.57755
AB171421	gi|110611175|ref|NM_000843.3 *Homo sapiens* glutamate receptor, metabotropic 6 (GRM6), mRNA	0.58226
AB096987	gi|239750853|ref|XR_079356.1 PREDICTED: *Homo sapiens* hypothetical protein LOC100130855 (LOC100130855), miscRNA	0.59997
AB049000	gi|209977116|ref|NM_080872.2 *Homo sapiens* unc-5 homolog D (*C. elegans*) (UNC5D), mRNA	0.60066
BB893759	gi|194018407|ref|NM_178148.2 *Homo sapiens* solute carrier family 35, member B2 (SLC35B2), mRNA	0.61849
AB056791	gi|162951883|ref|NM_014925.3 *Homo sapiens* R3H domain-containing 2 (R3HDM2), mRNA	0.64652
AB070176	gi|221307501|ref|NM_001143976.1 *Homo sapiens* WEE1 homolog (*S. pombe*) (WEE1), transcript variant 2, mRNA	0.64704
AB048962	gi|154146186|ref|NM_152634.2 *Homo sapiens* transcription elongation factor A (SII) N-terminal and central domain containing (TCEANC), mRNA	0.65673
DK578501	gi|24797073|ref|NM_033554.2 *Homo sapiens* major histocompatibility complex, class II, DP alpha 1 (HLA-DPA1), mRNA	0.65963

**Table 3:** Gene ontology of upregulated genes in Y-27632-treated TM cells.

Ontology	Term	Changed genes	Total genes	*P* value
Cellular component	Cell projection	31 (1)	331 (38)	0.0000306
Cellular component	Neuron projection	20 (6)	180 (28)	0.0000901
Cellular component	Cell projection part	17 (0)	142 (0)	0.000135
Biological process	Regulation of neurotransmitter levels	8 (0)	47 (2)	0.00148
Molecular function	Calcium channel regulator activity	4 (3)	11 (9)	0.00225
Cellular component	Presynaptic membrane	5 (5)	20 (20)	0.0024
Cellular component	Plasma membrane part	45 (0)	706 (2)	0.00259
Biological process	Synaptic transmission	16 (10)	170 (66)	0.00374
Molecular function	Channel regulator activity	5 (0)	23 (0)	0.00421
Biological process	Cellular nitrogen compound biosynthetic process	21 (0)	255 (0)	0.00443
Biological process	L-Glutamate import	3 (3)	6 (4)	0.00456
Cellular component	Platelet alpha granule	5 (1)	24 (2)	0.00472
Biological process	Transmission of nerve impulse	17 (0)	189 (3)	0.00563
Molecular function	Anion:cation symporter activity	4 (0)	15 (0)	0.00565
Molecular function	Sodium:dicarboxylate symporter activity	3 (3)	7 (7)	0.00585
Biological process	L-Amino acid import	3 (0)	7 (0)	0.00632
Biological process	Amino acid import	3 (0)	7 (0)	0.00632
Cellular component	Axon part	6 (0)	38 (2)	0.00665
Biological process	Regulation of mitotic cell cycle	8 (1)	62 (6)	0.00683
Molecular function	Anion transmembrane transporter activity	8 (0)	66 (5)	0.00813
Biological process	Cell-cell signaling	21 (4)	268 (74)	0.00841
Molecular function	High-affinity glutamate transmembrane transporter activity	2 (2)	2 (2)	0.00875
Cellular component	Cytoplasmic vesicle part	9 (0)	83 (2)	0.00918
Biological process	Deoxyribonucleoside triphosphate biosynthetic process	2 (0)	2 (0)	0.00923
Biological process	Response to calcium ion	5 (4)	28 (25)	0.00969
Molecular function	Rho guanyl-nucleotide exchange factor activity	5 (5)	29 (29)	0.00984
Biological process	Carboxylic acid transport	9 (0)	81 (1)	0.01005
Molecular function	Phosphatidylinositol binding	3 (3)	9 (9)	0.01012
Molecular function	Dicarboxylic acid transmembrane transporter activity	3 (0)	9 (0)	0.01012
Biological process	Organic acid transport	9 (0)	82 (0)	0.01077
Biological process	Glutamate metabolic process	3 (0)	9 (4)	0.01091
Biological process	Dicarboxylic acid transport	3 (3)	9 (7)	0.01091
Cellular component	Axoneme	4 (2)	19 (5)	0.01104
Molecular function	Structural constituent of cytoskeleton	5 (5)	31 (31)	0.01251
Cellular component	Cytoplasmic membrane-bounded vesicle lumen	4 (0)	20 (0)	0.01285
Cellular component	Platelet alpha granule lumen	4 (4)	20 (20)	0.01285
Cellular component	Dendritic spine	4 (4)	20 (20)	0.01285
Cellular component	Neuron spine	4 (0)	20 (0)	0.01285
Cellular component	Axon	9 (6)	89 (62)	0.01361
Cellular component	Neurofilament	2 (2)	3 (3)	0.01394
Molecular function	Antioxidant activity	5 (2)	32 (12)	0.01401
Biological process	Rho protein signal transduction	7 (1)	57 (15)	0.01403
Molecular function	Phenylalanine 4-monooxygenase activity	2 (2)	3 (3)	0.0142
Biological process	ER to Golgi vesicle-mediated transport	4 (4)	20 (20)	0.01457
Cellular component	Vesicle lumen	4 (0)	21 (1)	0.01484
Biological process	D-Amino acid transport	2 (0)	3 (0)	0.01497
Biological process	D-Aspartate import	2 (2)	3 (3)	0.01497
Biological process	D-Aspartate transport	2 (0)	3 (0)	0.01497
Biological process	Glutamate biosynthetic process	2 (2)	3 (3)	0.01497
Biological process	2′-Deoxyribonucleotide biosynthetic process	2 (0)	3 (0)	0.01497
Biological process	Fatty acid transport	4 (1)	21 (6)	0.01681
Molecular function	Ras guanyl-nucleotide exchange factor activity	5 (0)	34 (3)	0.01737
Biological process	Regulation of cell cycle process	6 (0)	47 (0)	0.01912
Biological process	Nucleoside triphosphate biosynthetic process	8 (1)	76 (3)	0.0195
Molecular function	Transporter activity	34 (8)	556 (147)	0.01993
Molecular function	Monocarboxylic acid binding	4 (0)	23 (0)	0.02002
Cellular component	Neurofilament cytoskeleton	2 (0)	4 (1)	0.02037
Biological process	Pyrimidine nucleoside triphosphate biosynthetic process	3 (0)	12 (0)	0.02063
Biological process	Pyrimidine nucleoside triphosphate metabolic process	3 (0)	12 (0)	0.02063
Molecular function	Oxidoreductase activity, acting on paired donors, with incorporation or reduction of molecular oxygen, reduced pteridine as one donor, and incorporation of one atom of oxygen	2 (1)	4 (2)	0.02075
Molecular function	Thioredoxin-disulfide reductase activity	2 (2)	4 (4)	0.02075
Biological process	Regulation of secretion	8 (0)	77 (0)	0.0208
Biological process	Neurotransmitter biosynthetic process	2 (2)	4 (4)	0.02186
Biological process	Tetrahydrobiopterin metabolic process	2 (1)	4 (1)	0.02186
Biological process	Deoxyribonucleoside triphosphate metabolic process	2 (0)	4 (1)	0.02186
Cellular component	Clathrin coat	4 (1)	24 (1)	0.02193
Biological process	Response to metal ion	8 (0)	78 (3)	0.02216
Cellular component	Transport vesicle membrane	3 (0)	13 (0)	0.02236
Biological process	Neurotransmitter metabolic process	3 (1)	13 (3)	0.02465
Biological process	Long-chain fatty acid transport	3 (2)	13 (4)	0.02465
Molecular function	Oxidoreductase activity, acting on sulfur group of donors	4 (0)	25 (0)	0.02549
Molecular function	Organic acid:sodium symporter activity	3 (0)	14 (0)	0.02707
Molecular function	Phosphoinositide binding	6 (2)	53 (30)	0.02738
Molecular function	Carboxylic acid binding	8 (0)	84 (5)	0.02754
Cellular component	MHC protein complex	4 (0)	26 (0)	0.02764
Molecular function	DNA topoisomerase type I activity	2 (2)	5 (5)	0.02831
Molecular function	Solute:sodium symporter activity	4 (0)	26 (0)	0.02853
Cellular component	Synapse	13 (10)	167 (116)	0.0289
Biological process	L-Amino acid transport	3 (0)	14 (1)	0.02906
Biological process	Cilium morphogenesis	3 (1)	14 (3)	0.02906
Cellular component	Secretory granule	8 (3)	86 (27)	0.02929
Biological process	Positive regulation of myeloid leukocyte differentiation	2 (0)	5 (0)	0.0298
Biological process	Glutamate catabolic process	2 (0)	5 (0)	0.0298
Biological process	Sulfate transport	2 (2)	5 (5)	0.0298
Biological process	Deoxyribonucleotide biosynthetic process	2 (0)	5 (2)	0.0298
Cellular component	Endomembrane system	26 (1)	416 (14)	0.03019
Cellular component	External side of plasma membrane	6 (5)	55 (49)	0.03035
Cellular component	Plasma membrane	64 (48)	1224 (935)	0.03044
Cellular component	Endocytic vesicle membrane	3 (3)	15 (12)	0.03078
Cellular component	Clathrin coated vesicle membrane	4 (0)	27 (3)	0.0308
Cellular component	Intrinsic to organelle membrane	8 (0)	87 (0)	0.03095
Biological process	Regulation of rho protein signal transduction	5 (5)	39 (29)	0.03101
Biological process	Neurotransmitter secretion	4 (3)	26 (11)	0.03114
Cellular component	Synapse part	10 (0)	117 (2)	0.03187
Biological process	Vitamin transport	3 (1)	15 (1)	0.03386
Molecular function	Symporter activity	6 (6)	57 (44)	0.03631
Molecular function	Syntaxin-1 binding	2 (2)	6 (6)	0.03678
Molecular function	Ion channel inhibitor activity	2 (1)	6 (2)	0.03678
Biological process	Cell communication	34 (4)	562 (40)	0.03717
Cellular component	Endocytic vesicle	4 (1)	29 (10)	0.03772
Biological process	Regulation of mitosis	4 (2)	28 (7)	0.03839
Biological process	Regulation of nuclear division	4 (0)	28 (0)	0.03839
Biological process	Response to inorganic substance	10 (1)	118 (8)	0.03843
Biological process	Phosphatidylcholine biosynthetic process	2 (2)	6 (5)	0.03869
Biological process	Interleukin-1 beta secretion	2 (0)	6 (0)	0.03869
Biological process	Interleukin-1 secretion	2 (0)	6 (0)	0.03869
Biological process	Regulation of interleukin-1 beta secretion	2 (0)	6 (0)	0.03869
Biological process	Regulation of interleukin-1 secretion	2 (0)	6 (0)	0.03869
Biological process	Cdc42 protein signal transduction	2 (1)	6 (3)	0.03869
Biological process	L-Phenylalanine catabolic process	2 (2)	6 (6)	0.03869
Biological process	L-Phenylalanine metabolic process	2 (0)	6 (0)	0.03869
Biological process	Tyrosine metabolic process	2 (0)	6 (1)	0.03869
Biological process	Multicellular organismal aging	2 (1)	6 (2)	0.03869
Biological process	Aspartate transport	2 (0)	6 (3)	0.03869
Biological process	Negative regulation of transforming growth factor beta receptor signaling pathway	2 (2)	6 (6)	0.03869
Biological process	Neurofilament cytoskeleton organization	2 (1)	6 (5)	0.03869
Molecular function	Chloride ion binding	4 (4)	29 (29)	0.0389
Biological process	L-Glutamate transport	3 (0)	16 (7)	0.03904
Biological process	Platelet activation	3 (2)	16 (11)	0.03904
Biological process	Positive regulation of secretion	5 (0)	42 (1)	0.03976
Molecular function	Substrate-specific transporter activity	28 (0)	463 (0)	0.04034
Biological process	Nucleotide biosynthetic process	12 (0)	151 (7)	0.04052
Cellular component	Microtubule basal body	3 (3)	17 (17)	0.04062
Molecular function	Calcium-dependent protein binding	3 (3)	17 (17)	0.04164
Biological process	Anion transport	7 (0)	73 (10)	0.04185
Molecular function	Solute:cation symporter activity	5 (0)	44 (0)	0.04201
Biological process	Purine nucleoside triphosphate biosynthetic process	7 (0)	74 (0)	0.0443
Biological process	Acidic amino acid transport	3 (0)	17 (0)	0.04461
Biological process	Pyrimidine nucleoside metabolic process	3 (0)	17 (1)	0.04461
Biological process	Pyrimidine nucleotide biosynthetic process	3 (0)	17 (4)	0.04461
Cellular component	MHC class II protein complex	3 (3)	18 (18)	0.04607
Molecular function	Oxidoreductase activity, acting on sulfur group of donors, NAD, or NADP as acceptor	2 (0)	7 (2)	0.04608
Molecular function	Fatty acid transporter activity	2 (1)	7 (3)	0.04608
Molecular function	Channel inhibitor activity	2 (0)	7 (1)	0.04608
Molecular function	DNA topoisomerase activity	2 (1)	7 (2)	0.04608
Biological process	Regulation of cytokine production	7 (0)	75 (0)	0.04684
Molecular function	NADP or NADPH binding	3 (3)	18 (17)	0.04721
Biological process	Nucleobase, nucleoside, and Nucleotide biosynthetic process	12 (0)	157 (0)	0.04725
Biological process	Nucleobase, nucleoside, nucleotide, and nucleic acid biosynthetic process	12 (0)	157 (0)	0.04725
Biological process	G1 phase	2 (1)	7 (1)	0.04845
Cellular component	Lamellipodium	4 (4)	32 (32)	0.04965
Cellular component	Transport vesicle	4 (1)	32 (11)	0.04965

**Table 4:** Gene ontology of downregulated genes in Y-27632-treated TM cells.

Ontology	Term	Changed genes	Total genes	*P* value
Molecular function	Endonuclease activity, active with either ribo- or deoxyribonucleic acids and producing 5′-phosphomonoesters	2 (0)	13 (0)	0.00279
Biological process	Antigen processing and presentation of peptide or polysaccharide antigen via MHC class II	2 (2)	16 (14)	0.00421
Cellular component	MHC class II protein complex	2 (2)	18 (18)	0.00606
Molecular function	D-Dopachrome decarboxylase activity	1 (1)	1 (1)	0.01065
Molecular function	Glucose 1-dehydrogenase activity	1 (1)	1 (1)	0.01065
Molecular function	Glucose-6-phosphate dehydrogenase activity	1 (1)	1 (1)	0.01065
Molecular function	Deoxyribonuclease I activity	1 (1)	1 (1)	0.01065
Molecular function	3′-Phosphoadenosine 5′-phosphosulfate transmembrane transporter activity	1 (1)	1 (1)	0.01065
Biological process	Olfactory behavior	1 (1)	1 (1)	0.01091
Biological process	Positive regulation of dopamine receptor signaling pathway	1 (1)	1 (1)	0.01091
Biological process	Regulation of dopamine receptor signaling pathway	1 (0)	1 (0)	0.01091
Biological process	3′-Phosphoadenosine 5′-phosphosulfate transport	1 (1)	1 (1)	0.01091
Biological process	Negative regulation of neuron apoptosis	2 (2)	28 (28)	0.01148
Cellular component	MHC protein complex	2(0)	26(0)	0.01171
Molecular function	Toxin binding	1 (1)	2 (2)	0.01594
Molecular function	C2H2 zinc finger domain binding	1 (1)	2 (2)	0.01594
Molecular function	Dopachrome isomerase activity	1 (1)	2 (2)	0.01594
Molecular function	6-Phosphogluconolactonase activity	1 (1)	2 (2)	0.01594
Molecular function	Hedgehog receptor activity	1 (1)	2 (2)	0.01594
Molecular function	Dihydrofolate reductase activity	1 (1)	2 (2)	0.01594
Molecular function	Purine nucleoside transmembrane transporter activity	1 (0)	2 (1)	0.01594
Biological process	Cytolysis by symbiont of host cells	1 (0)	2 (0)	0.01632
Biological process	Cytolysis of cells in other organism during symbiotic interaction	1 (0)	2 (0)	0.01632
Biological process	Cytolysis of cells of another organism	1 (0)	2 (0)	0.01632
Biological process	Disruption by symbiont of host cells	1 (0)	2 (0)	0.01632
Biological process	Hemolysis by symbiont of host erythrocytes	1 (1)	2 (2)	0.01632
Biological process	Hemolysis of cells in other organism	1 (0)	2 (0)	0.01632
Biological process	Hemolysis of cells in other organism during symbiotic interaction	1 (0)	2 (0)	0.01632
Biological process	Killing by symbiont of host cells	1 (0)	2 (0)	0.01632
Biological process	Maintenance of mitochondrion location	1 (1)	2 (2)	0.01632
Biological process	Modification by organism of cell membrane in other organism during symbiotic interaction	1 (0)	2 (0)	0.01632
Biological process	Modification by symbiont of host cell membrane	1 (0)	2 (0)	0.01632
Biological process	Modification by symbiont of host cellular component	1 (0)	2 (0)	0.01632
Biological process	Modification by symbiont of host structure	1 (0)	2 (0)	0.01632
Biological process	Modification of cellular component in other organism during symbiotic interaction	1 (0)	2 (0)	0.01632
Biological process	Modification of structure of other organism during symbiotic interaction	1 (0)	2 (0)	0.01632
Biological process	Caveola assembly	1 (1)	2 (2)	0.01632
Biological process	Membrane raft assembly	1 (0)	2 (0)	0.01632
Biological process	Positive regulation of G-protein coupled receptor protein signaling pathway	1 (0)	2 (1)	0.01632
Biological process	Chromatin-mediated maintenance of transcription	1 (1)	2 (2)	0.01632
Biological process	Positive regulation of gene expression, epigenetic	1 (0)	2 (0)	0.01632
Biological process	ncRNA catabolic process	1 (0)	2 (0)	0.01632
Biological process	rRNA catabolic process	1 (1)	2 (2)	0.01632
Biological process	Purine nucleoside transport	1 (0)	2 (1)	0.01632
Biological process	Striated muscle cell differentiation	2 (1)	38 (5)	0.0199
Biological process	Antigen processing and presentation	2 (2)	38 (32)	0.0199
Molecular function	Copper ion binding	2 (2)	39 (38)	0.01996
Molecular function	Endodeoxyribonuclease activity, producing 5′-phosphomonoesters	1 (0)	3 (1)	0.0212
Cellular component	Membrane	24 (17)	2769 (1768)	0.02168
Biological process	Maintenance of organelle location	1 (0)	3 (0)	0.02171
Biological process	Melanin biosynthetic process	1 (1)	3 (3)	0.02171
Biological process	Melanin metabolic process	1 (0)	3 (0)	0.02171
Biological process	Endoplasmic reticulum calcium ion homeostasis	1 (1)	3 (1)	0.02171
Biological process	Multicellular organismal water homeostasis	1 (0)	3 (0)	0.02171
Biological process	Renal water homeostasis	1 (1)	3 (1)	0.02171
Biological process	Positive regulation of Rac protein signal transduction	1 (1)	3 (3)	0.02171
Biological process	Apoptosis	7 (3)	522 (189)	0.0221
Biological process	Programmed cell death	7 (0)	525 (2)	0.02273
Cellular component	Extrinsic to internal side of plasma membrane	1 (1)	3 (3)	0.02347
Cellular component	Spectrin	1 (1)	3 (3)	0.02347
Molecular function	Endonuclease activity	2 (2)	43 (30)	0.02378
Molecular function	Nucleoside transmembrane transporter activity	1 (0)	4 (1)	0.02643
Biological process	Regulation of neuron apoptosis	2 (0)	45 (2)	0.02693
Biological process	Detection of visible light	1 (1)	4 (1)	0.02706
Biological process	Chemosensory behavior	1 (0)	4 (3)	0.02706
Biological process	Protein maturation by protein folding	1 (1)	4 (4)	0.02706
Biological process	Cellular chaperone-mediated protein complex assembly	1 (1)	4 (2)	0.02706
Biological process	Mitochondrial outer membrane translocase complex assembly	1 (1)	4 (4)	0.02706
Biological process	Outer mitochondrial membrane organization	1 (0)	4 (0)	0.02706
Biological process	Glycine biosynthetic process	1 (1)	4 (2)	0.02706
Cellular component	Membrane part	21 (0)	2325 (1)	0.02771
Biological process	Cellular membrane organization	4 (0)	212 (30)	0.02909
Biological process	Membrane organization	4 (0)	212 (0)	0.02909
Biological process	Neuron apoptosis	2 (0)	48 (3)	0.03021
Biological process	Neuron death	2 (0)	48 (0)	0.03021
Biological process	Muscle cell differentiation	2 (0)	49 (1)	0.03134
Biological process	Disruption of cells of other organism during symbiotic interaction	1 (0)	5 (0)	0.03239
Biological process	Killing of cells in other organism during symbiotic interaction	1 (0)	5 (0)	0.03239
Biological process	Water homeostasis	1 (0)	5 (1)	0.03239
Biological process	Endoplasmic reticulum organization	1 (1)	5 (5)	0.03239
Biological process	Membrane raft organization	1 (0)	5 (1)	0.03239
Biological process	Pinocytosis	1 (1)	5 (2)	0.03239
Biological process	Nucleoside transport	1 (0)	5 (2)	0.03239
Biological process	Response to light stimulus	2 (0)	51 (8)	0.03364
Biological process	Cell death	7 (0)	578 (68)	0.03597
Biological process	Vesicle-mediated transport	5 (2)	336 (120)	0.03622
Biological process	Death	7 (0)	579 (0)	0.03626
Biological process	Positive regulation of signaling pathway	3 (0)	133 (0)	0.03743
Biological process	Modification by host of symbiont morphology or physiology	1 (0)	6 (0)	0.03769
Biological process	ER overload response	1 (1)	6 (5)	0.03769
Biological process	Regulation of Rac protein signal transduction	1 (0)	6 (1)	0.03769
Molecular function	Actin binding	3 (3)	139 (121)	0.0394
Cellular component	HOPS complex	1 (1)	6 (6)	0.04072
Molecular function	Intramolecular oxidoreductase activity, transposing C=C bonds	1 (0)	7 (0)	0.04196
Biological process	Detection of light stimulus	1 (0)	7 (0)	0.04296
Biological process	Metabotropic glutamate receptor signaling pathway	1 (1)	7 (4)	0.04296
Biological process	Regulation of synaptic transmission, GABAergic	1 (1)	7 (2)	0.04296
Cellular component	Internal side of plasma membrane	1 (0)	7 (4)	0.04641
Molecular function	Scavenger receptor activity	1 (1)	8 (8)	0.04708
Biological process	Interaction with symbiont	1 (0)	8 (1)	0.0482
Biological process	Modification by symbiont of host morphology or physiology	1 (0)	8 (0)	0.0482
Biological process	Chaperone-mediated protein complex assembly	1 (0)	8 (4)	0.0482
Biological process	Positive regulation of Ras protein signal transduction	1 (0)	8 (4)	0.0482
Biological process	Positive regulation of small GTPase-mediated signal transduction	1 (0)	8 (0)	0.0482
Biological process	Synaptic transmission, GABAergic	1 (0)	8 (1)	0.0482
Biological process	Actin filament capping	1 (1)	8 (6)	0.0482
Biological process	Pentose-phosphate shunt	1 (1)	8 (7)	0.0482
Biological process	Calcium ion transport	2 (1)	63 (41)	0.04876
Cellular component	Intrinsic to membrane	17 (0)	1867 (20)	0.0491
